# Randomized Controlled Ferret Study to Assess the Direct Impact of 2008–09 Trivalent Inactivated Influenza Vaccine on A(H1N1)pdm09 Disease Risk

**DOI:** 10.1371/journal.pone.0086555

**Published:** 2014-01-27

**Authors:** Danuta M. Skowronski, Marie-Eve Hamelin, Gaston De Serres, Naveed Z. Janjua, Guiyun Li, Suzana Sabaiduc, Xavier Bouhy, Christian Couture, Anders Leung, Darwyn Kobasa, Carissa Embury-Hyatt, Erwin de Bruin, Robert Balshaw, Sophie Lavigne, Martin Petric, Marion Koopmans, Guy Boivin

**Affiliations:** 1 British Columbia Centre for Disease Control, Vancouver, British Columbia, Canada; 2 University of British Columbia, Vancouver, British Columbia, Canada; 3 Centre Hospitalier Universitaire de Québec [University Hospital Centre of Québec], Québec, Canada; 4 Laval University, Québec, Canada; 5 Institut National de Santé Publique du Québec [National Institute of Health of Québec], Québec, Canada; 6 Institut universitaire de cardiologie et pneumologie de Québec, Québec, Québec, Canada; 7 Public Health Agency of Canada, Winnipeg, Manitoba, Canada; 8 Department of Medical Microbiology, University of Manitoba, Winnipeg, Manitoba, Canada; 9 Canadian Food Inspection Agency, Winnipeg, Manitoba, Canada; 10 Laboratory for Infectious Disease Research, Diagnostics and Screening, Centre for Infectious Disease Control (CIDC), Rijksinstituut voor Volksgezondheid en Milieu (RIVM) [National Institute of Public Health and the Environment], Bilthoven, The Netherlands; 11 Simon Fraser University, Burnaby, British Columbia, Canada; 12 Viroscience Department, Erasmus MC, Rotterdam, The Netherlands; Centers for Disease Control and Prevention, United States of America

## Abstract

During spring-summer 2009, several observational studies from Canada showed increased risk of medically-attended, laboratory-confirmed A(H1N1)pdm09 illness among prior recipients of 2008–09 trivalent inactivated influenza vaccine (TIV). Explanatory hypotheses included direct and indirect vaccine effects. In a randomized placebo-controlled ferret study, we tested whether prior receipt of 2008–09 TIV may have directly influenced A(H1N1)pdm09 illness. Thirty-two ferrets (16/group) received 0.5 mL intra-muscular injections of the Canadian-manufactured, commercially-available, non-adjuvanted, split 2008–09 Fluviral or PBS placebo on days 0 and 28. On day 49 all animals were challenged (Ch0) with A(H1N1)pdm09. Four ferrets per group were randomly selected for sacrifice at day 5 post-challenge (Ch+5) and the rest followed until Ch+14. Sera were tested for antibody to vaccine antigens and A(H1N1)pdm09 by hemagglutination inhibition (HI), microneutralization (MN), nucleoprotein-based ELISA and HA1-based microarray assays. Clinical characteristics and nasal virus titers were recorded pre-challenge then post-challenge until sacrifice when lung virus titers, cytokines and inflammatory scores were determined. Baseline characteristics were similar between the two groups of influenza-naïve animals. Antibody rise to vaccine antigens was evident by ELISA and HA1-based microarray but not by HI or MN assays; virus challenge raised antibody to A(H1N1)pdm09 by all assays in both groups. Beginning at Ch+2, vaccinated animals experienced greater loss of appetite and weight than placebo animals, reaching the greatest between-group difference in weight loss relative to baseline at Ch+5 (7.4% vs. 5.2%; p = 0.01). At Ch+5 vaccinated animals had higher lung virus titers (log-mean 4.96 vs. 4.23pfu/mL, respectively; p = 0.01), lung inflammatory scores (5.8 vs. 2.1, respectively; p = 0.051) and cytokine levels (p>0.05). At Ch+14, both groups had recovered. Findings in influenza-naïve, systematically-infected ferrets may not replicate the human experience. While they cannot be considered conclusive to explain human observations, these ferret findings are consistent with direct, adverse effect of prior 2008–09 TIV receipt on A(H1N1)pdm09 illness. As such, they warrant further in-depth investigation and search for possible mechanistic explanations.

## Introduction

During spring-summer 2009, several observational studies from Canada reported that prior receipt of the 2008–09 trivalent inactivated influenza vaccine (TIV) was associated with increased risk of medically-attended, laboratory-confirmed A(H1N1)pdm09 illness, with estimated risk or odds ratios of 1.4–2.5 compared to those unvaccinated [1]. This increased risk was not apparent among vaccinated people when comparing hospitalized to community cases [1], and observational studies in other settings showed contradictory results, including increased [2]–[5], null [6]–[9] or protective [10], [11] effects from vaccination. Hypotheses to explain findings from Canada initially focused on methodologic (observational designs) or product-specific (domestically-manufactured vaccine) considerations. However, a randomized-controlled trial (RCT) in Hong Kong spanning November 2008 to October 2009 also showed significantly increased relative risk (2.58) among children who had received a different manufacturer’s 2008–09 TIV product (Vaxigrip, Sanofi Pasteur, Lyon, France) [12], [13].

Previous ferret studies have also shown mixed results although none have demonstrated 2008–09 TIV to have been protective against A(H1N1)pdm09[14]–[19]. Two small ferret studies reported no TIV effect on virus replication in nasal or lung specimens [14], [15] but, where clinical outcomes have been assessed, several studies have shown consistent albeit non-significant trend toward greater weight loss and worsening of severity indicators in vaccinated ferrets [16]–[19]. All of these ferret studies to date, however, have suffered from small sample size, typically comparing ≤5 animals per group in total.

Mechanistic hypotheses to explain increased A(H1N1)pdm09 risk among prior TIV recipients have included both direct and indirect vaccine effects [1]. The direct effect hypothesis postulates that seasonal vaccine may directly influence host resistance to pandemic virus infection and/or replication whereas the indirect hypothesis proposes that seasonal vaccine may block the more robust, complex and cross-protective immunity otherwise afforded by seasonal virus infection thereby indirectly increasing the risk of pandemic illness. Here we report on a randomized, blinded, placebo-controlled ferret study to test whether the commercially-available TIV predominantly used in Canada in 2008–09 may have directly influenced A(H1N1)pdm09 disease risk.

## Materials and Methods

### Ethics Statement

Animal procedures were approved by the Institutional Animal Care Committee of Laval University according to the guidelines of the Canadian Council on Animal Care (protocol 2011055).

### Overview

**Figure 1:**
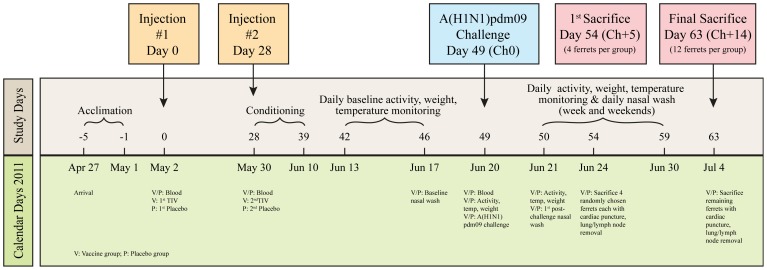
Study protocol. Randomized blinded placebo-controlled experiment of Canadian manufactured, commercially-available 2008–09 trivalent inactivated influenza vaccine (TIV: Fluviral) on A(H1N1)pdm09 disease risk in ferrets.

Experimental procedures were conducted at the Laval University animal care facility in Québec between April 27 and July 4, 2011 (**Figure 1**). Animals were housed two per cage in the same room and permitted food and water *ad libitum*.

The primary outcome of this study was weight loss. Sample size was assigned to test differences in proportionate weight loss from baseline following infection with pandemic H1N1 virus. Based on 80% power and 2-sided alpha of 0.05 to detect mean difference in weight loss relative to baseline of 5% in the placebo group and 10% in the vaccine group, and given standard deviation (SD) of 4–5%, 12–17 ferrets per group would be required. We used 16 ferrets per group.

### Intervention

Thirty-two male ferrets were randomly assigned to receive 0.5 mL intra-muscular injection of either 2008–09 TIV (“vaccine”) or PBS (“placebo”) on days 0 and 28. Study personnel and investigators remained blinded to vaccine/placebo assignment throughout experimental procedures and assays. GlaxoSmithKline (GSK Fluviral; manufactured in Laval, Québec, Canada) and Sanofi Pasteur (Vaxigrip; manufactured in Lyon, France) supplied approximately 75% and 25%, respectively, of the seasonal split TIV distributed in Canada during the 2008–09 season. In this experiment we therefore used the dominant Canadian manufactured, commercially-available, non-adjuvanted GSK sodium deoxycholate split-antigen Fluviral containing the three WHO-recommended vaccine components: A/Brisbane/59/2007(H1N1)-like, A/Brisbane/10/2007(H3N2)-like, and B/Florida/4/2006(Yamagata)-like [20]. Manufacturers substituted the egg-adapted reassortant strains A/Brisbane/59/2007(H1N1) IVR-148 (hereafter “IVR-148”) and A/Uruguay/716/2007(H3N2) NYMC X-175C (hereafter “X-175C”) as considered antigenically-equivalent vaccine components.

The potency of all three vaccine strains of the post-expiry but cold-chain maintained 2008–09 Fluviral lot that was used was confirmed by single radial immuno-diffusion (SRID) testing by the Center for Biologics Evaluation and Research, United States (US) Food and Drug Administration (FDA) [21]. US specifications require ≥27 µg/mL hemagglutinin (HA) for each antigen (dose 0.5 mL) based on the mean of three tests, with additional requirements around the standard deviation (SD). As last assessed in February 2012, for each antigen, mean HA content for the TIV lot used remained ≥30 µg/mL with SDs within required specifications.

### Challenge and Sacrifice

On day 49, animals were lightly anesthetized and challenged (Ch0) intra-nasally with 250 µL (4.5logTCID50/mL) [22], [23] of a Québec A(H1N1)pdm09 isolate (A/Québec/144147/2009; propagated thrice in vitro in Madin-Darby canine kidney (MDCK) cells; GenBank: FN434457–FN434464) (**Table S1**), administered half-volume per nostril. Four randomly-selected ferrets per group were sacrificed on day 54 (i.e. 5 days post-challenge: Ch+5) and the remainder sacrificed on day 63 (i.e. 14 days post-challenge: Ch+14).

### Antibody Response

Pre-shipment sera collected between March 14 and 24, 2011 and serum samples from anesthetized animals collected on days 0 (May 2, 2011), 28, 49 (Ch0), 54 (Ch+5) and 63 (Ch+14) were assessed for antibody to TIV components and A(H1N1)pdm09 by haemagglutination inhibition (HI), microneutralization (MN), nucleoprotein (NP)-based ELISA and HA1-based protein microarray assays.

#### Hemagglutination inhibition and microneutralization – antigens and analyses

Reference viruses used in the HI and MN assays were obtained from Canada’s Influenza Reference Laboratory, the National Microbiology Laboratory (NML), Winnipeg, and passaged in MDCK cells two to three times (**Table S1**). MDCK-passaged viruses were tested by both HI and MN against reference ferret anti-serum provided by the NML, confirming antigenic integrity was maintained relative to the WHO-recommended reference viruses. Viral hemagglutinin (HA) and neuraminidase (NA) were also sequenced to assess amino acid (AA) identity relative to WHO reference viruses and the actual vaccine components selected by the manufacturer (**Table S1 and Table S2**). Compared to respective vaccine strains, passaged assay viruses showed complete HA and NA identity for seasonal H1N1 and ≥98% for H3N2. Compared to challenge virus, passaged A(H1N1)pdm09 assay virus also showed ≥98% HA and NA identity. Percent identity was reduced as expected when comparing seasonal to pandemic H1 strains (72–73% in the HA1 protein).

HI and MN assays were conducted in duplicate according to established protocols [24] as detailed below and the individual result assigned as the geometric mean titer (GMT) of duplicate values. Summary HI and MN serologic statistics were compared including the number meeting threshold titers of 10 and of 40. Changes in titers from baseline (day 0) to days 28, 49 (Ch0), and 54 (Ch+5) or 63 (Ch+14) were assessed through sero-conversion, group GMTs with 95% confidence intervals (CI) and group GMT ratios (GMTR).

#### Haemagglutination inhibition methods

In preparation for the HI assay, sera were treated with receptor destroying enzyme (Accurate Chemical & Scientific, NY) to remove non-specific inhibitors of agglutination, and further hemadsorbed with 50% turkey erythrocytes (Lampire Biologic Laboratories, Pennsylvania). Sera were serially diluted beginning at 1:10 with phosphate buffered saline and 25 µL of each serum dilution was reacted with 25 µL of antigen (infected MDCK cell lysate supernatant) containing 4 HA units of virus for 30 minutes. To each mixture 50 µL of 0.5% turkey erythrocytes were added, and after mixing, the preparations were incubated for 30 minutes. Results were recorded by photography. The HI titer was designated as the inverse of the highest dilution at which detectable HI activity was still present. Influenza B virus was used in the HI assay in its ether-treated, inactivated form. Briefly, the clarified influenza B virus cell lysates were each mixed by agitation with an equal volume of diethyl ether. The mixture was kept at 4°C for 15 minutes to allow for the phases to separate. The top ether layer was aspirated and nitrogen gas bubbled through the virus preparation to remove residual ether, and the preparation was then used in the HI assay.

#### Microneutralization methods

For MN assay, 50% tissue culture infectious dose (TCID50) viral titers were determined on MDCK cells. The sera were treated with receptor destroying enzyme (Accurate Chemical & Scientific, NY) and serially diluted in serum-free medium (MegaVir, HyClone, Utah) beginning at 1:10. To each 50 µL dilution, 100 infectious units of virus were added. The plates were incubated for 2 hours at 37°C to allow for virus antibody interaction. The contents of each well were then transferred onto microtiter plates with confluent monolayers of MDCK cells. After 3 hours of further incubation at 37°C, the medium in each well was removed and replaced with fresh MegaVir medium containing 2 µg/mL L-1-tosylamido-2-phenylethyl chloromethyl ketone (TPCK)-treated trypsin. The plates were further incubated at 37°C and monitored for cytopathic effects on days 3 and 5. The MN titer was defined as the inverse of the serum dilution immediately preceding the wells with cytopathic effects.

#### ELISA and HA1-based protein microarray methods

Competitive ELISA was conducted on sera using a commercially-available NP-based influenza A antibody test kit from IDEXX Laboratories, Inc. (Switzerland) [25]. Competitive ELISA antibody values were calculated as sample optical density (OD) divided by negative control OD with ratios <0.60 considered positive and mean values with 95%CI displayed; smaller ELISA ratios denote higher antibody levels.

An HA1-based protein microarray serological assay was conducted for study and non-study antigens listed in **Table S3**, performed as previously described [26] with adaptation for detection of ferret antibodies. Serum samples were tested in a single 1:10 dilution in Blotto containing 0.1% Surfact Ampt (both Thermo Fisher Scientific Inc., Rockford, USA). All incubation steps were one hour at 37°C. Recombinant HA1 proteins were spotted in duplicate and incubated with 70 µL Blotto followed by 70 µL of diluted ferret serum and then two-step conjugation by incubation with 70 µL mouse anti-mustelid IgG (Antibodies-online, Aachen, Germany) followed by 70 µL Dylight 649-conjugated goat anti-mouse IgG (Jackson Immunoresearch, Baltimore pike, USA) both in Blotto containing 0.1% Surfact Ampt. After washing and drying, the slides were scanned using a Powerscanner (Tecan, Männedorf, Switzerland) and signals quantified using a Scanarray scanner (Perkin Elmer, Waltham, USA). Individual spot signals were valued with correction by subtraction for per spot background fluorescence, truncated at zero where signal was below background. Final individual per ferret values were assigned as the average of duplicates, log_10_-transformed after imputing a value of 0.1 for any zero values and compared for each antigen by study group and day.

### Clinical Monitoring

Activity, rectal temperature, appetite and weight were recorded daily from days 42 through 46 and then from challenge (day 49; Ch0) until sacrifice. Baseline reference weight per ferret was computed as the average of day 42 to 46 and Ch0 weights. Activity was scored 1–5: one being the most alert and playful; two, alert but playful only if induced; three, alert but not playful (stays in hiding place); four, neither alert nor playful and five was the humane endpoint. Appetite was scored as usual, diminished or no appetite.

### Nasal Wash and Lung Virus Quantification

A(H1N1)pdm09 virus titers were assessed in nasal wash pre-challenge day 46 and daily post-challenge (Ch+1) until sacrifice and in whole right lung homogenates at each scheduled sacrifice. Virus titers were determined by standard plaque assays using St6GalI-expressing MDCK cells [27], [28], expressed as log_10_-transformed plaque-forming units (pfu) per mL.

### Lung Histopathology and Immuno-histochemistry

Paraffin tissue sections were prepared from whole left lung of ferrets for histo-pathology assessment at each scheduled sacrifice. Inflammation was graded on six indicators (bronchial/endobronchial, peribronchial, perivascular, interstitial, pleural and intra-alveolar), each scored as: 0 (normal), 1 (mild), 2 (moderate) or 3 (marked) for a maximum combined score of 18 [28]. Vascular congestion and pulmonary edema were similarly scored.

Lung immuno-histochemistry was undertaken to identify cells with virus antigen. Paraffin tissue sections were quenched for 10 minutes in aqueous 3% H_2_O_2_ then pre-treated with proteinase K for 15 minutes. A 1:10,000 dilution of mouse monoclonal antibody to influenza A nucleoprotein (F26NP9; in-house) was applied for one hour. Sections were visualized using horseradish peroxidase-labelled polymer, Envision®+system (anti-mouse) (Dako, USA), reacted with the chromogen, diaminobenzidine and counter-stained with Gill’s hematoxylin.

### Lung Cytokine Response

Change in lung cytokine mRNA gene expression was assessed by relative quantitative PCR (qPCR) with RNA extracted from 140 µL of right lung homogenate using the QIAamp viral RNA mini-kit. Reverse transcription was performed using the High Capacity RNA-to-cDNA Kit (Applied Biosystems) on 0.5 µg of the total RNA following manufacturer’s protocol. qPCR was performed using the TaqMan® Gene Expression Master Mix (ABI) with primers designed to target published cytokine sequences [29]–[31] from Mustela putorius furo mRNA sequences at a final concentration of 0.25 µM. Assays were run on the StepOne Plus (ABI) with the following conditions: 50°C–2 minutes; 95°C–10 minutes; followed by 40 cycles of 95°C–15 seconds; and 60°C–1 minute. Fold-change was calculated using the delta-delta Ct method [32] with uninfected ferrets as reference and GAPDH as endogenous control.

### Statistical Methods

Chi-square or Fisher’s exact test were used to compare categorical variables and t-test or Wilcoxon methods for continuous variables. Weight, nasal virus titers, and HA1 protein microarray values were analysed via contrasts in a mixed-effects linear model (group, visit and their interaction, with repeated measurements on each animal). For protein microarray values, analyses were repeated after excluding statistical outliers without substantively affecting main conclusions. Inflammatory scores by individual ferret are presented and combined inflammatory scores and cytokine values at Ch+5 versus Ch+14 were compared via contrasts in a two-way ANOVA model (group, scheduled sacrifice day and their interaction). Observed p<0.05 are described as significant; no attempt is made to correct for multiple inference.

## Results

### Antibody Response

**Figure 2:**
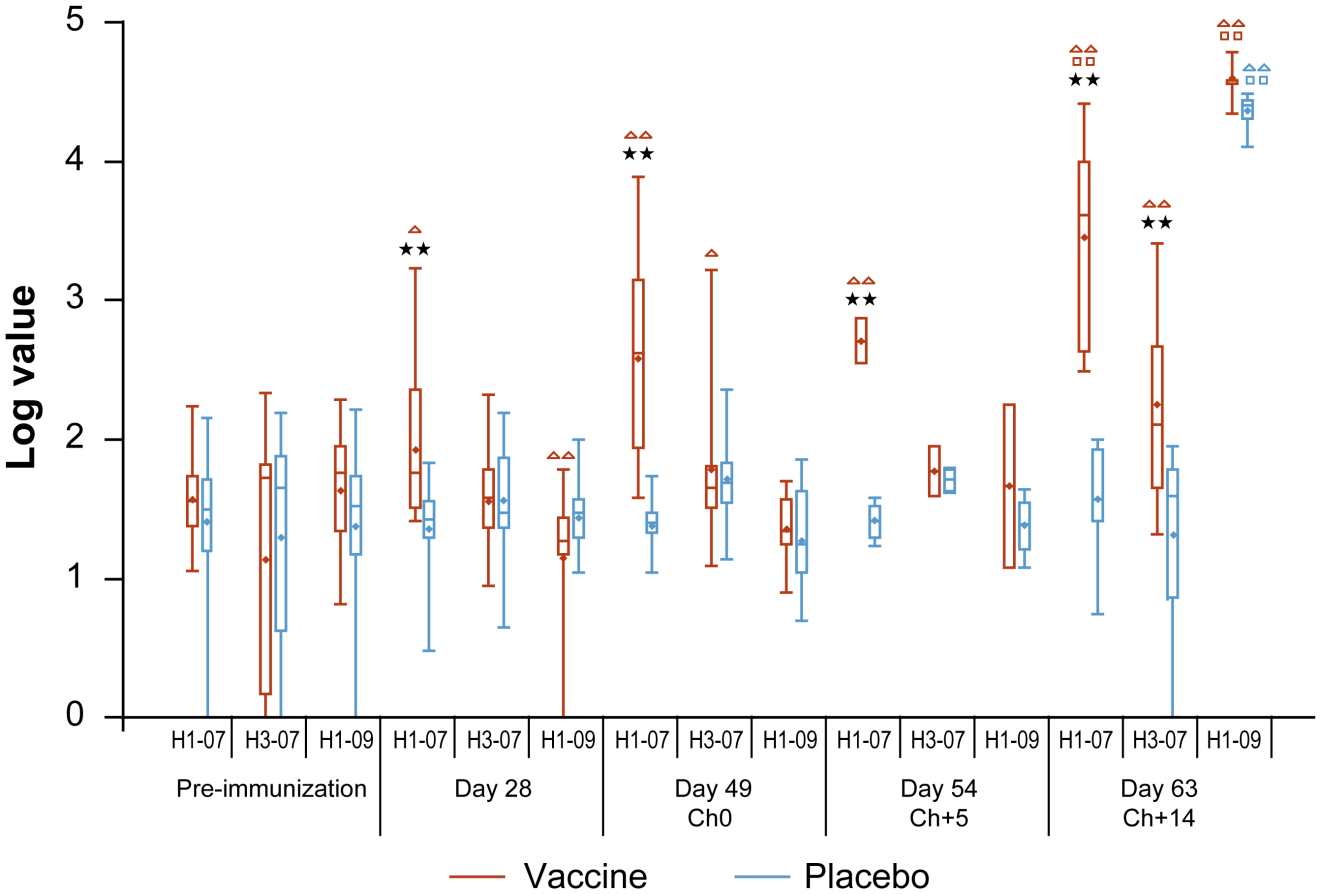
HA1 microarray serological values by study antigens, group and day. Box plots display median (dash) and mean (dot) of log_10_-transformed HA1 protein microarray signal values. The box extends to the 25^th^/75^th^ percentiles and whiskers extend to minimum/maximum values. H1-07 indicates A/Brisbane/59/2007 (H1N1)-like; H3-07 indicates A/Brisbane/10/2007 (H3N2)-like; H1-09 indicates A/California/7/2009 (H1N1)pdm09-like (**Table S3;** grey-shaded). Sample size as follows: Pre-immunization Vaccine = 15, Placebo = 16 (3 ferrets each per group pre-shipment serum was substituted owing to insufficient day 0 available); Day 28 Vaccine = 14, Placebo = 15; Day 49 Vaccine = 12, Placebo = 11; Day 54 Vaccine = 2, Placebo = 4; Day 63 Vaccine = 9, Placebo = 8. **indicates statistical significance at p<0.01 and *indicates statistical significance at p<0.05 in comparing vaccine to placebo group at the designated time point. ΔΔ indicates statistical significance at p<0.01 and Δ indicates statistical significance at p<0.05 in comparing values within study groups at days 28, 49, 54 and 63 relative to pre-immunization, colour coded by vaccine (red) or placebo (blue). □□ indicates statistical significance at p<0.01 and □ indicates statistical significance at p<0.05 in comparing day 63 to day 49 within groups, colour coded per above by study group.

Individual HI, MN and ELISA assay results are displayed concurrently for pre-shipment, day 0, 28, 49 and 54/63 time points, for the four vaccinated and four placebo animals sacrificed at Ch+5 in **Table S4**, and for the 12 animals per group sacrificed at day 63 in **Table S5** for vaccinated animals and **Table S6** for placebo animals. Summary ELISA results are shown in **Table 1** and summary microarray results for study antigens in **Figure 2** with study and non-study antigen cross-reactive responses shown in **Figure S1**. Summary HI and MN statistics are shown in **Table S7 and Table S8**, respectively.

**Table 1:** Influenza A antibody results by study day and group based on ELISA assay.

	Number ELISA antibody positive/Number of sera tested^a^		Mean ELISA antibody result (95% confidence interval)^b^	
Time point (N randomizedper group)	Vaccine	Placebo	Vaccine	Placebo
Pre-Shipment (16)	0/16	0/16	1.18 (1.09–1.27)	1.18 (1.08–1.28)
Day 0 (16)	0/16^c^	0/16^c^	1.03 (0.98–1.08)	1.02 (0.96–1.09)
Day 28 (16)	ND	ND	ND	ND
Day 49/Ch0 (16)	10/14	0/13	0.51 (0.35–0.66)	1.03 (0.99–1.06)
Day 54/Ch+5 (4)	4/4	0/4	0.21 (0.09–0.33)	1.03 (0.89–1.15)
Day 63/Ch+14 (12)	10/10	9^d^/11	0.17 (0.09–0.25)	0.46 (0.39–0.53)

**Competitive Nucleoprotein-based IDEXX ELISA (Influenza A antibody). Ratios <0.60 classified as positive; ratios ≥0.60 classified as negative.**

ND = Not done; IDEXX Inc. = Commercial ELISA assay (note the lower the ratio, the greater the antibody detected); Ch = challenge.

a Where numbers tested differ from the number randomized per group in parentheses at the specified time point it is because insufficient sera remained for testing of all animals.

b Number of sera tested by group shown in adjacent columns.

c One day 0 serum in each group was insufficient for ELISA testing and substituted with pre-shipment values for these ferrets. Excluding these ferrets (leaving n = 15 per group) gives ELISA ratios of 1.04 (0.99–1.09) and 1.05 (1.01–1.09) for vaccine and placebo groups, respectively.

d Two sera belonging to placebo group close to serologic threshold for positivity with ratios of 0.61.

All animals were confirmed by NP-based ELISA to be influenza A naïve at pre-shipment and day 0. By day 49 there was significant ELISA antibody rise following immunization in the vaccine group, with further antibody rise evident by day 54 (Ch+5) after A(H1N1)pdm09 challenge in vaccinated animals but not until day 63 (Ch+14) in placebo ferrets (**Table 1** **, Table S4, Table S5, Table S6**). Ultimately, total influenza A ELISA antibody was significantly higher among vaccine compared to placebo animals at both scheduled sacrifices (**Table 1**).

Protein microarray results were consistent with ELISA but in addition showed vaccine-induced HA1 antibody to the seasonal H1 antigen, for which values were significantly higher in vaccinated animals relative to pre-immunization and compared to placebo from day 28, most pronounced from day 49 after the first TIV dose (i.e. three weeks after two-dose vaccine series completion) (**Figure 2**). There was significant rise in antibody relative to pre-immunization for the H3N2 vaccine component at day 49, but only relative to the placebo group at day 63 (i.e. five weeks after vaccine series completion). Slight but significant decrease in A(H1N1)pdm09 antibody was evident at day 28 post-immunization compared to baseline but not thereafter or relative to placebo, calling into question its clinical relevance. Significant rise in antibody to A(H1N1)pdm09 relative to pre-immunization was evident at day 63 in both the vaccine and placebo groups. Of interest, infection with A(H1N1)pdm09 induced antibody by day 63 not only to itself but also to closely related 1918 H1 antigen in both study groups (significantly higher for the latter in the vaccinated) (**Figure S1**). Furthermore, at day 63 among vaccinated but not among placebo animals, A(H1N1)pdm09 challenge induced significantly greater cross-reactivity to non-study (1977, 1999) H1 variants to which the animals had never been exposed, evident relative to pre-immunization, to the placebo group and to pre-challenge at day 49 (**Figure S1**). Increase following A(H1N1)pdm09 challenge among the vaccinated was evident at day 63 for other non-H1, non-vaccine antigens (H2, H3) relative to pre-immunization but not relative to placebo.

All animals were also shown by HI and MN assays to be influenza naïve at pre-shipment (**Table S4, Table S5, Table S6**). More variability in HI than MN antibody titers was evident thereafter. Overall, however, the significant rise in vaccine-induced antibody shown by ELISA and HA1 protein microarray by day 49 was not evident by HI or MN assays except among a few of the vaccinated ferrets (**Table S4** **, Table S5**). Conversely, ferrets in both groups showed substantial neutralizing antibody to A(H1N1)pdm09 at day 63 (**Table S5, Table S6**) with very high GMTs and GMTRs relative to baseline, slightly (but non-significantly) higher in the placebo compared to the vaccine group by both HI and MN (**Table S7, Table S8**).

### Clinical Findings

Average baseline weight was comparable between the vaccine (0.96 Kg) and placebo (0.97 Kg) groups (p = 0.87). Beginning at Ch+2, more vaccinated animals showed diminished or no appetite compared to placebo recipients (7/16 versus 3/16; p = 0.25) with the greatest between-group difference at Ch+5 (12/16 versus 6/16; p = 0.07), that resolved by Ch+10 (1/12 vs. 0/12; p = 1.0). The greatest between-group difference for no appetite was at Ch+6 (7/12 vs. 1/12; p = 0.03).

**Figure 3:**
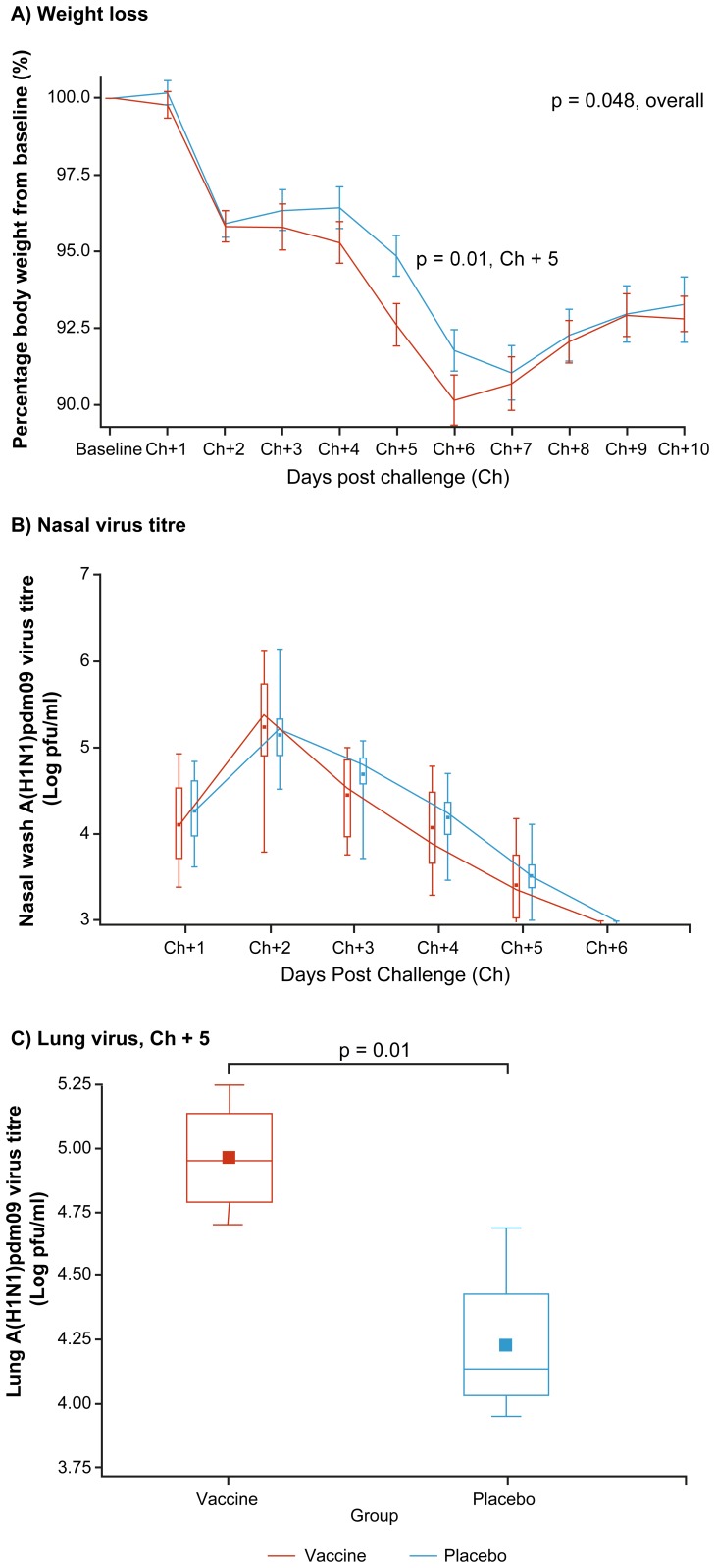
Clinical outcomes including weight loss, nasal wash and lung virus titers by study group and day. Clinical outcomes are displayed including: (A) Mean percentage weight relative to baseline by study group and day, with standard errors. (B) Nasal wash virus titers by study group and day. (C) Lung homogenate virus titers at day 5 post-challenge. Box plots (B and C) display mean (dot) and median (line) virus titres as log pfu/mL. Per usual, the box extends to the 25^th^/75^th^ percentiles and whiskers extend to minimum/maximum values. Ch refers to challenge day and Ch+1, Ch+2 etc indicate day post-challenge (i.e. day one post-challenge, day two post-challenge etc). Ch+5 indicates day five post-challenge on which four animals per group were randomly selected for sacrifice. Statistically significant between-group differences are as specified.

Weight loss from baseline was greater in the vaccine than placebo group, evident beginning at Ch+2 and marginally significant across the full study period (% weight loss p = 0.048; absolute loss p = 0.06) (**Figure 3A**). The greatest between-group difference in percentage weight loss from baseline was at Ch+5 (7.4% vs. 5.2%; p = 0.01) (**Figure 3A**) on which day 4/16 (25%) vaccinated versus 1/16 (6%) placebo animals had lost 10% or more of their body weight compared to baseline (p = 0.3) (**Table S4, Table S5, Table S6**). Consistent with random selection, the same pattern was evident regardless of sacrifice day, with mean percentage weight loss from baseline at Ch+5 of 6.9% for the vaccine group and 4.6% for the placebo group among animals selected for sacrifice at Ch+5 and 7.6% and 5.3%, respectively, among animals sacrificed instead at Ch+14. Animals had comparable scores of one for alertness/playfulness at all time points except from Ch+2–8 for which activity levels were marginally worse (scored as two) in all animals in both groups. Temperature patterns did not differ between vaccine and placebo groups with a peak in mean/median temperatures at Ch+2 of 40.0°C and 40.1°C, respectively.

### Nasal Wash and Lung Virus Quantification

Nasal A(H1N1)pdm09 titers did not differ significantly between groups over time (p = 0.37). Nasal virus titers rose more steeply between Ch+1–2 in the vaccine versus placebo group (mean difference in log-titres 1.14 versus 0.88 pfu/mL; p = 0.25) and then fell more steeply at Ch+2–3 (mean difference in log-titres 0.80 versus 0.46 pfu/mL; p = 0.01) (**Figure 3B**).

Lung A(H1N1)pdm09 titers at Ch+5 were significantly higher in the vaccine versus placebo group (log-mean 4.96 versus 4.23 pfu/mL; p = 0.01) (**Figure 3C**). Neither group had detectable virus in the lung at Ch+14.

### Lung Histopathology

**Figure 4:**
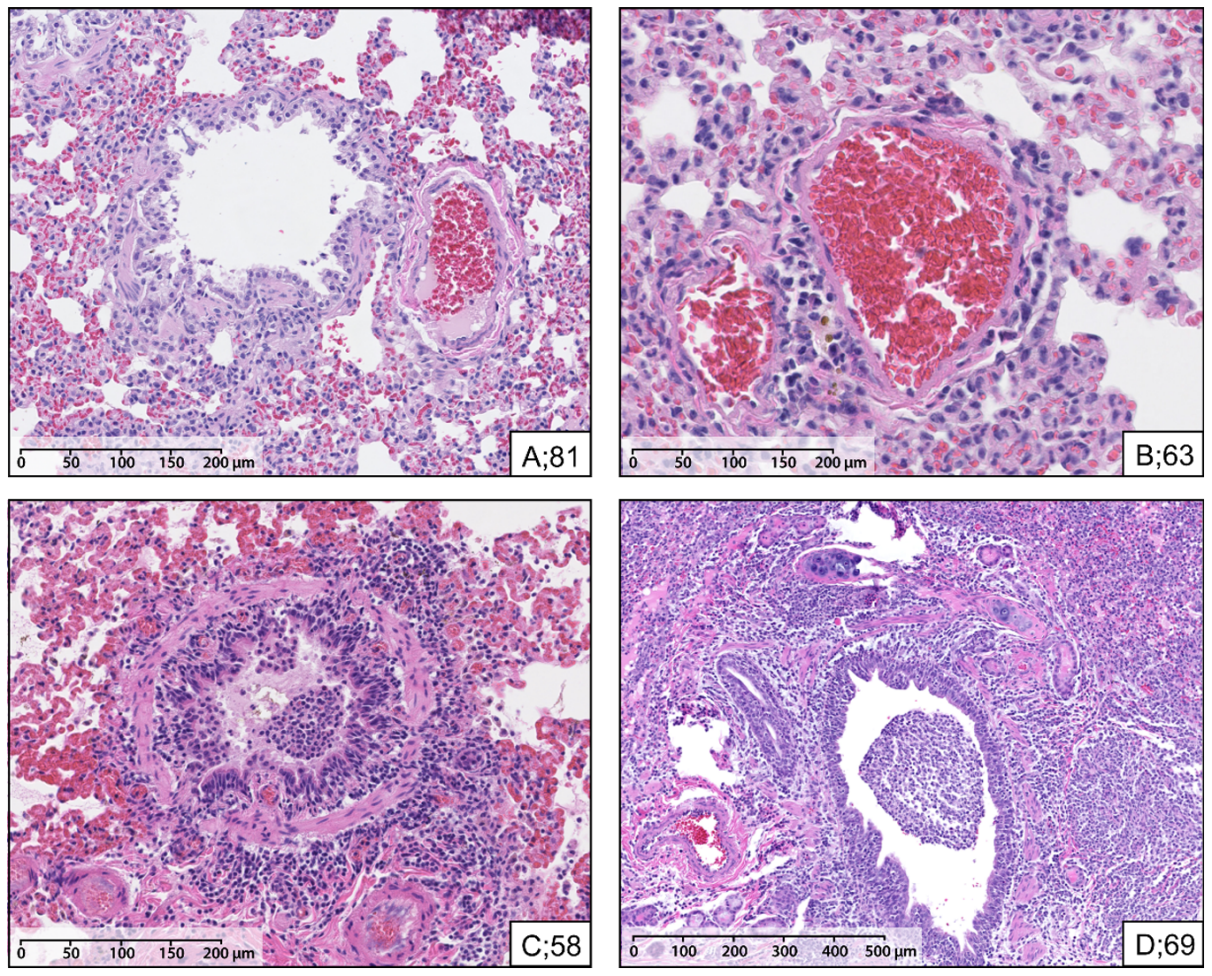
Ferret lung histology at day 5 post-challenge (Ch+5). Salient lung histologic features (hematoxylin eosin stain micrometric scale in lower left of each panel) including vaccine and placebo ferrets with highest and lowest combined Ch+5 inflammatory scores within their group: (A) Ferret #81 (placebo; inflammatory score 0.5) indicating very mild/minimal peri-bronchial inflammation; (B) Ferret #63 (vaccinated; inflammatory score 0.5) indicating very mild/minimal peri-vascular inflammation; (C) Ferret #58 (placebo; inflammatory score 4.0) indicating moderate bronchial and mild peri-bronchial/peri-vascular inflammation; (D) Ferret #69 (vaccinated; inflammatory score 11.5) indicating severe bronchopneumonia. The increased micrometric scale in panel D reflects that the pathologic changes are marked and diffuse, best rendered at low magnification, whereas the mild to moderate and more focal changes in panels A to C necessitate photomicrographs at higher magnification. Corresponding histopathology scores are shown in **Table S9**.

At Ch+5, animals sacrificed from the vaccine group had higher combined mean/median lung inflammatory scores than placebo animals (5.8/5.5 versus 2.1/2.0), a difference that did not reach statistical significance (p = 0.051) (**Table S9**). Two of four vaccinated animals showed a combined lung inflammatory score of ≥10 at Ch+5 compared to none of the four placebo animals (maximum score = 4) (**Table S9**). Salient histologic features are shown in **Figure 4** for animals of both groups with the highest and lowest combined Ch+5 inflammatory scores, illustrating the severe bronchopneumonia that was evident in half of the vaccinated but none of the placebo animals. The increased micrometric scale in panel D reflects that the pathologic changes are marked and diffuse, best rendered at low magnification, whereas the mild to moderate and more focal changes in panels A to C necessitate photomicrographs at higher magnification.

Compared to Ch+5, combined mean lung inflammatory score was significantly lower in the vaccine group among animals sacrificed at Ch+14 (5.8 vs. 1.8; p = 0.01) whereas this showed little change over time in the placebo group (2.1 vs. 2.4; p = 0.84) with significant interaction between scheduled sacrifice day and group (p = 0.048). Combined mean/median lung inflammatory scores were not significantly different between the vaccine versus placebo group at Ch+14 (1.8/1.2 versus 2.4/1.5).

In both the vaccine and placebo groups at Ch+5, influenza antigen was detected by immuno-staining in bronchial and bronchiolar epithelium. Within alveolar walls, most of the positive cells were identified as pneumocytes using double immuno-labelling [not shown]. Occasional cells had the morphology of macrophages. No viral antigen was detected in animals at Ch+14.

### Lung Cytokines

**Figure 5:**
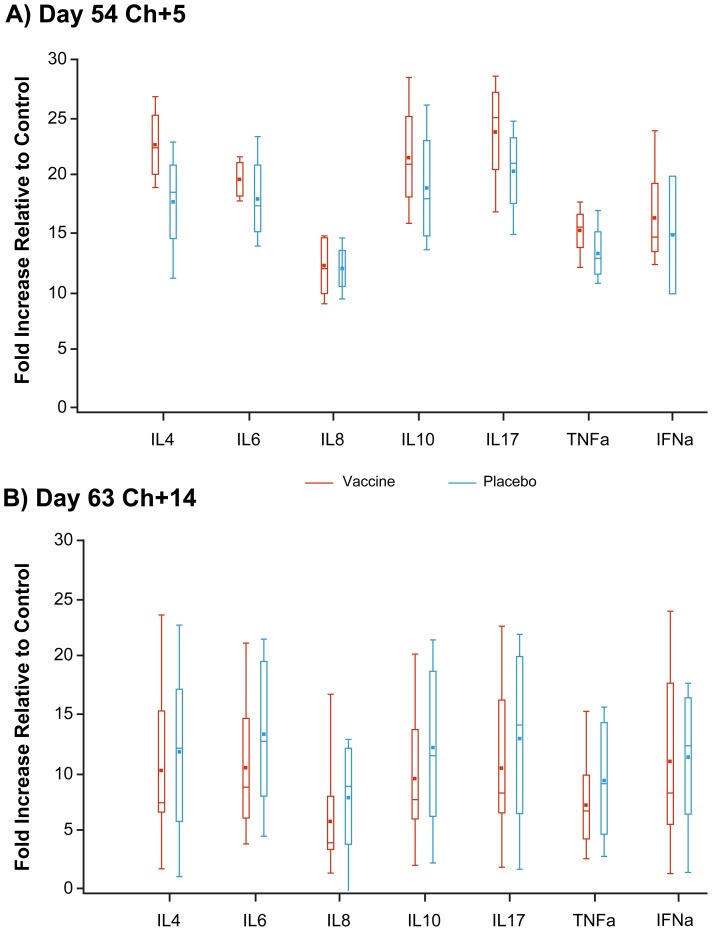
Lung cytokine values at days 5 and 14 post-challenge with A(H1N1)pdm09 by study group. Per usual, box plots display mean (dot) and median (dash) virus titers with box extending to the 25^th^/75^th^ percentiles and whiskers extending to minimum/maximum values. Cytokine values relative to control are displayed at (A) day 5 post challenge (Ch+5) on which four animals per group were randomly selected for sacrifice and (B) day 14 post-challenge (Ch+14) on which the remaining 12 animals per group were sacrificed. Thus, at Ch+5, n = 4 (except IFN alpha for which n = 2 for placebo group) and at Ch+14, n = 12.

IFN gamma was below the limits of detection in both groups at both scheduled sacrifices. Other lung cytokines were consistently but non-significantly higher in the vaccine versus placebo group at Ch+5. All cytokine values were lower in both groups at Ch+14, consistently but non-significantly lower in the vaccine animals. The Ch+5 versus Ch+14 difference was statistically significant for all vaccine group cytokines (all p≤0.02) except IFN alpha (p>0.05) whereas differences were not significant in the placebo group, except for IL17 (p = 0.046). Overall, the interaction between scheduled sacrifice day and study group was not significant for any cytokine (**Figures 5A and 5B**).

## Discussion

During spring-summer 2009, several observational studies from Canada reported that prior recipients of 2008–09 TIV experienced approximately two-fold increased risk of medically-attended, laboratory-confirmed A(H1N1)pdm09 illness [1]. Recognizing that all observational designs are susceptible to methodological bias, gold standard RCT analysis would typically be considered essential in clarifying such unexpected findings. In Hong Kong, an RCT of the 2008–09 TIV (Vaxigrip) already underway had shown similar association among vaccinated children with significant relative risk of 2.58 (increased to 2.74 with adjustment for seasonal infection) [12], [13]; however, during follow-up RCT using 2009–10 Vaxigrip and spanning August 2009 to December 2010, the same investigators instead reported significant protective effects [33]. Both RCTs lacked sufficient power for analysis based on virologically-confirmed infection so that conclusions were drawn instead from less reliable serologically-defined outcomes [12], [33]. Further RCT test of the association in humans has now become practically impossible given that, since fall 2010, all seasonal TIV routinely contains protective, homologous A(H1N1)pdm09 antigen [20]. We therefore undertook RCT evaluation of the possible direct effects of prior heterologous TIV receipt on A(H1N1)pdm09 disease risk in ferrets as the ideal alternate model of human influenza infection [34].

Although none of the animals became moribund or so severely ill as to require euthanasia, ferrets immunized with two doses of 2008–09 TIV did show significantly worse clinical, virologic and pathological features following pandemic H1N1 infection compared to placebo recipients. As originally powered to show, vaccinated animals experienced significantly greater weight loss relative to baseline following infection, maximally different from placebo at Ch+5. Nasal wash titers did not differ, but vaccinated animals showed significantly higher lung virus titers. Consistent with lung virus findings, lung inflammation was also increased more than 2.5-fold in vaccinated compared to placebo animals at Ch+5 although with fewer animals sacrificed on that day the difference fell just short of statistical significance. Inflammatory indicators were, however, significantly higher at Ch+5 compared to Ch+14 in the vaccinated animals and showed no change across that period in placebo recipients. Lung cytokines showed a similar pattern. Although illness activity levels appeared similar between groups based on categories of induced playfulness, these may have been of insufficient resolution to reflect clinical differences in lung disease, manifest otherwise through significant loss of appetite and weight.

By day 14 post-challenge animals in both groups had recovered. In human studies, a doubling of the risk of medically-attended pandemic H1N1 illness was observed among 2008–09 TIV recipients, but an increase in the risk of hospitalization was not shown [1]. This was broadly interpreted to suggest increased risk of *acquiring* infection *per se* whereas here we report increased disease severity among influenza-naïve, systematically-infected ferrets. As such, our findings in ferrets may not replicate the experience in humans. It is worth noting, however, that the source population in the human observational studies was patients seeking medical care [1]. Although illness severity among outpatient visits was not specifically compared between vaccinated and unvaccinated participants, subjects had experienced influenza-like illness severe enough to prompt medical consultation within one week of illness onset, and that outcome was significantly increased among the vaccinated. In that regard, the pattern of acute worsening of A(H1N1)pdm09 illness during the first week of infection in vaccinated ferrets, followed by subsequent recovery by day 14, is consistent with increased outpatient but not hospitalization risk observed in vaccinated humans. As such, this ferret RCT suggests that earlier findings from observational studies in humans cannot be dismissed on the basis of methodological bias alone and that direct mechanistic explanations should be sought. Whether these findings may be product-specific or may also apply to other TIV products has yet to be separately assessed in follow-up studies.

To date, hypotheses about biological mechanisms to explain increased A(H1N1)pdm09 risk among prior TIV recipients have included both direct and indirect vaccine effects [1]. Indirect mechanisms include the infection block hypothesis whereby effective seasonal vaccine may prevent the more robust and complex cross-protective immunity against heterologous viruses afforded by seasonal infection, such as through cell-mediated responses to conserved internal virus components [1], [35], [36]. Other epidemiological investigators have favoured this hypothesis, or related variations (such as temporary immunity hypothesis) [33], [37], but in Appendix G of our original publication [1] we demonstrated these indirect hypotheses to be insufficient and implausible to fully explain a doubling of risk, requiring as they do unreasonably high estimates of infection attack rates, infection-induced cross-protection, and TIV effectiveness [1], [13]. To test whether increased risk in vaccinees may have occurred without invoking infection block mechanisms we specifically designed the current ferret experiment without the intermediary of heterologous seasonal influenza infection. As such, we cannot rule out an additive role for indirect vaccine effects mediated through infection block mechanisms, but show that direct vaccine effects are likely to have at least contributed to our previous findings.

Possible direct vaccine effects include antibody-dependent enhancement (ADE) whereby virus uptake by cells is enhanced in the presence of low-level, cross-reactive, non-neutralizing antibodies, best described for dengue [1], [38], [39]. A possible role for cross-reactive antibodies in explaining severe A(H1N1)pdm09 manifestations in otherwise healthy adults, and in archived lung sections from fatal adult cases during the 1957 H2 pandemic has previously been suggested [40]. Another recent study has reported an association between higher ratios of cross-reactive ELISA versus neutralizing antibody titers early during A(H1N1)pdm09 infection and more severe illness [41]. Enhanced respiratory disease in vaccinated swine has also been reported following challenge with A(H1N1)pdm09 or other heterologous, homosubtypic H1 viruses that do not share cross-reactive neutralizing antibodies [42]–[45]. As in our ferret experiment, clinical worsening in vaccinated swine was evident at two to five days post-challenge [43], [44] and was correlated with elevated pro-inflammatory cytokine responses in the lung [44]. Unlike the current ferret or prior human studies, however, these swine studies used adjuvanted whole virion vaccine that was additionally heterosubtypic for the neuraminidase surface protein (i.e. N2 versus N1), the relevance of which is uncertain. ADE is classically associated with enhanced virus uptake in macrophages or other Fc-receptor-bearing cells, demonstrated in vitro for influenza [46]–[49] and more recently also specifically for A(H1N1)pdm09 in the presence of heterologous human anti-sera [50]. In our vaccinated ferrets, higher lung virus titers were observed, but immuno-histochemistry could not distinguish affected cells of the lung in vaccine versus placebo animals, and macrophages were not predominant in either group. More recently in swine, however, heterologous antibody has been shown to enhance A(H1N1)pdm09 infection of other mammalian (MDCK) cells, described in the context of fusion-enhancing cross-reactive anti-HA2 stalk antibodies and absent neutralizing antibodies targeting the HA1 globular head [51], [52].

Our experiment was unable to further elucidate these putative immunologic mechanisms. We identified significant vaccine-induced influenza A antibody rise by ELISA and confirmed this to include anti-HA1 antibody response to the seasonal H1N1 TIV component. There was also rapid and significant increase in influenza A ELISA antibody following A(H1N1)pdm09 challenge among vaccinated but not placebo ferrets sacrificed at Ch+5 but HA1-based and neutralizing antibodies to A(H1N1)pdm09 were not evident until Ch+14 in either group. Lower neutralizing antibody to A(H1N1)pdm09 even at Ch+14 among vaccinated versus placebo ferrets, although not statistically significant, is consistent with human immunogenicity trials showing blunting of pandemic H1N1 vaccine-induced responses in association with prior seasonal vaccine receipt [53]–[56]. Conversely, pandemic H1N1 immunization has been observed to boost pre-existing heterosubtypic antibody which may also be consistent with our Ch+14 findings for the H3N2 TIV component and the broad boosting of cross-reactive antibodies to other antigenically-distant H1 variants observed by protein microarray in vaccinated but not placebo animals [57]. Although protein microarray did not show cross-reactive A(H1N1)pdm09 antibody prior to Ch+14, it was not designed to detect the sort of anti-HA2 stalk antibodies highlighted above in association with severe disease in vaccinated swine [51]. Ultimately, therefore, we are unable to discern whether the rise in Ch+5 ELISA antibody in vaccinated animals suggests early cross-reactive, non-neutralizing antibody to A(H1N1)pdm09 or further antibody increase to TIV antigens 4 weeks after their second dose (or both) although microarray indicates the latter certainly contributed.

T-cell hypo-responsiveness may be an alternate explanation compatible with a hypothesis of direct vaccine effect. This phenomenon has been reported in same-season influenza vaccine booster-dose studies [58] with parallels also in the allergy literature suggesting peptide-induced T-cell hypo-responsiveness beginning at 2–8 weeks and lasting up to 40 weeks [59]. However, while interferon-gamma was below detectable limits in both groups, lung cytokines in vaccinated ferrets were otherwise consistently (but non-significantly) higher compared to placebo animals at Ch+5, notably including the Th2 IL4 [60], pro-inflammatory IL17 [61], [62] and regulatory IL10 [63] cytokines. IL17 has been implicated as super-inducer of neutrophil infiltration and acute lung immuno-pathology following influenza infection [62], with counteractive dampening interactions by IL10 [63]. In an earlier Canadian ferret experiment in which animals administered a single dose of 2008–09 TIV also experienced worse A(H1N1)pdm09 illness, IL6 in nasal wash was substantially raised in the Fluviral group and IL10 significantly in the Flumist group, with disease enhancement suggested in both vaccine groups compared to controls [16]. We did not assess nasal wash cytokines or Flumist and were not statistically powered to explore cytokine differences, but lung IL6 and IL10 were also both non-significantly raised at Ch+5 in our Fluviral versus placebo ferrets. All cytokine values were then lower at Ch+14 in the absence of lung pathology. However, none of the between-group cytokine differences at either time point were statistically significant.

There are limitations to this study. Although ferrets are considered the ideal animal model for human influenza infection, there are anticipated differences in immunologic and clinical aspects of immunization, infection and illness responses (timing, dosing and intensity) across species. Overall patterns may be compared but ferret studies do not support precise quantification of actual risk in humans. The greater likelihood of more severe disease based on several clinical indicators (weight loss, lung virus titers) among vaccinated compared to unvaccinated ferrets may not replicate the greater likelihood of medically-attended A(H1N1)pdm09 illness we previously reported in vaccinated humans. In using influenza-naïve, systematically infected ferrets there are clear differences from the human experience with respect to pre-conditions (influenza exposure history and immunologic context), process (infection acquisition), and other relevant parameters (vaccine immunogenicity, clinical outcomes and monitoring). Clinical relevance of the differences we report between vaccinated and placebo ferrets is ultimately best interpreted in the context of our study objectives assigned in follow up to the prior human observations we reported. The main objective of the ferret study was to assess through randomized, controlled design whether prior receipt of 2008–09 TIV may have had direct, adverse effects on A(H1N1)pdm09 illness, specifically powered related to weight loss. Although we cannot more precisely elucidate the underlying mechanisms involved, the current ferret study supports the hypothesis of direct vaccine effect. Taken together with prior human and swine studies, these findings represent a signal that warrant further investigation and better understanding though they cannot be considered conclusive.

The most prominent concern in this ferret study may relate to our failure to show neutralizing antibody response to vaccine, an issue we therefore consider in detail. We observed some greater albeit low-level variability in antibody titers by HI, a non-functional assay, than by microneutralization or NP-based ELISA. Intra- and inter-laboratory variability in antibody assay results is well-recognized [64], [65] and we thus interpret findings in the context of combined HI, MN, and ELISA results, overall indicating our animals were naïve at pre-shipment and baseline. Vaccine-induced HI and MN responses were not evident thereafter but significant vaccine-induced antibody rise was shown by both NP-based ELISA and HA1-based protein microarray assays, the latter also shown only at high serum/low antibody concentrations (i.e. testing dilution of 1:10). Although SRID testing of the expired 2008–09 vaccine lot that we used still met standard HA potency requirements for annual commercial vaccine approval by the FDA [21], we cannot rule out other unrecognized vaccine changes with time that may have been influential.

MDCK-passaged viruses used in our HI and MN assays were antigenically equivalent to reference strains and amino acid sequencing showed they were also identical in their HA and NA to the 2008–09 H1N1 vaccine component. This argues against such linear differences to explain the suboptimal neutralizing responses we measured; however, we cannot rule out other conformational changes to protein structure in the original vaccine comprised of (sodium-deoxycholate) disrupted and inactivated virus. The H3N2 virus used in our assays was also antigenically equivalent to the WHO-recommended reference virus with which it shared ≥98% HA antigenic site amino acid identity. However, we did not conduct antigenic testing in relation to the actual vaccine component used by manufacturers and cannot rule out differences between assay and vaccine viruses in the suboptimal vaccine responses we measured (**Table S1 and S2**). Protein micro-array also showed variable low-level response to the H3N2 component among immunized ferrets at day 49, significantly greater than placebo and compared to pre-immunization only at day 63 (i.e. five weeks after the second vaccine dose). Whether the latter was due to further antibody rise with time following immunization or cross-reactive response following A(H1N1)pdm09 challenge is unknown; however, similar effect was not observed relative to placebo for other non-vaccine H3 subtype viruses included in the microarray, suggesting H3N2 vaccine response.

Thresholds for defining antibody response are also anticipated to vary across species and assays; these have not been cross-correlated/−validated in ferrets for the various antigens and assays we used. In fact, sero-protective thresholds have not yet been established for ferrets by any assay. However, failure to induce a robust HI or MN antibody response to inactivated influenza vaccine in naïve ferrets has long been recognized, after single or several doses, in the absence of prior infection or adjuvant for seasonal, novel or pandemic vaccines [66]–[73]. In order to replicate more closely the observations in humans, we used a commercially available, non-adjuvanted 2008–09 Fluviral lot that had been administered in Canada. It is of note that in human studies conducted with the same product in 2008–09 (pediatric) [74], 2009–10 (elderly) [75] and in a mouse study conducted in 2010–11 [21], Fluviral also induced suboptimal HI and/or MN responses to the same seasonal A/Brisbane/59/2007-like H1N1 vaccine antigen. For example, in the pediatric trial including infants and toddlers 6–23 months of age similarly naïve to influenza as were our ferrets, the same schedule of two 0.5 mL doses of a thimerosal-free version of the 2008–09 Fluviral induced significantly lower H1N1 antibody responses than even half that volume (0.25 mL) per dose of Vaxigrip [74]. Despite double the HA content per dose, GMTs at four weeks post-immunization with the 2008–09 Fluviral (39.8 [95%CI: 27.6–57.5]) were significantly lower than with the 2008–09 Vaxigrip (100.2 [95%CI: 59.8–168.0]), lower still when Fluviral was administered at the conventional 0.25 mL dose typically given to children this age (30.0 [95%CI: 20.5–43.8]) [74]. A similar pattern, though less pronounced, was also observed with the H3N2 component [74]. The reasons for diminished immunogenicity of the Canadian vaccine are unknown although authors of the pediatric trial proposed more complete clearance of intact virus among other possible explanations.

In human observational studies, the 2008–09 TIV was still shown to be protective overall against homologous seasonal influenza [1]. Spring-summer 2009 observations of increased risk of heterologous A(H1N1)09 illness were identified six or more months after TIV receipt. In that regard, lower vaccine-induced antibody titers in ferrets at our three-week post-immunization A(H1N1)pdm09 challenge time point may better replicate end-of-season antibody conditions when vaccinated humans were exposed to A(H1N1)pdm09 virus. A prior ferret study to assess the same 2008–09 Fluviral also suggested disease enhancement but was able to induce homologous HI antibody response to the H1N1 component with mean antibody titre exceeding 100 within two weeks of a single 0.5 mL vaccine dose [16], higher even than induced in the pediatric study population cited above. Such variability in serologic responses may reflect lot-to-lot or laboratory differences. Not knowing the precise mechanisms involved in vaccine-associated enhanced respiratory disease, and unable to exactly know or replicate the human immunologic context in spring-summer 2009, we did not adjust the human vaccine formulation, dose or schedule to force higher ferret vaccine responses. Instead we focused on clinical parameters, recording the observed effects according to standard immunization practice.

In general, ferret studies to date have suffered from small sample size and insufficient power [14]–[19], [76]. Our study was powered for clinical (percentage weight loss) comparison and follow-up to 14 days post-challenge. Failure to reach statistical significance for other consistent indicators should not prompt their dismissal but should stimulate further investigation. It may be argued that lung findings at Ch+5 were chance occurrences among few ferrets poorly-representative of the full group experience. However, animals in both groups were randomly and blindly selected for Ch+5 sacrifice, the comparison of baseline and Ch+5 characteristics showed no significant within-group differences according to scheduled endpoint, and Ch+5 lung findings in vaccinated animals (higher virus titers and lung inflammation) were consistent with overall clinical patterns (greater loss of appetite and weight). Nevertheless, future experiments should be powered with more animals to specifically examine these early acute clinical, immunologic, and pathologic findings and explore their possible mechanisms in greater detail.

We assessed only influenza-naïve animals whereas most humans, other than young children, will have prior potentially cross-attenuating influenza infection history. The use of influenza-naïve animals in previous swine [42]–[45], [51], [52] and the current ferret studies may be relevant to the increased severity highlighted in vaccinated animals but to a lesser extent noted with the association in people. Further experiments are needed to explore nuances related to infection and/or immunization history which additionally and variously complicate the human experience. In a recent publication, disease enhancement was included among possible hypotheses to explain greater 2009 pandemic H1 morbidity in the Americas compared to Australia, New Zealand or Europe, with reference to findings in vaccinated swine interpreted ecologically in the context of regional differences in prior heterologous seasonal H1N1 virus circulation; given findings in vaccinated ferrets and swine, however, regional differences in prior heterologous seasonal H1N1 vaccine (i.e. TIV) coverage may also be relevant to consider [77]. Of note, mechanisms such as ADE, if explanatory, require a precise balance of low-level, cross-reactive, non-neutralizing antibody to be manifest [1], [38], [39], a particular but sliding immunologic scale that may not have been captured in all animals or humans at the time of A(H1N1)pdm09 exposure. A spectrum of illness is anticipated with any infection process and a greater likelihood of severity does not require that all exposed individuals experience that outcome. However, this additional immunologic complexity related to ADE, if involved, may have contributed to the variability in clinical outcomes we observed among vaccinated ferrets and to the variability in reporting the association in humans. Our experiment assessed the unique context of heterologous but homosubtypic pandemic H1N1 challenge. It has been suggested that original antigenic sin as an aspect of the cross-reactive, non-neutralizing antibody required for ADE applies when antigenic differences of less than 33–42% exist across related but distinct prime-boost strains [38]; amino acid differences in the HA1 between the 2008–09 seasonal and 2009 pandemic H1 antigens were within this range (72–73% similarity; **Table S2**) with much closer homology (92%) across the HA2 (18 amino acid differences across 222 residues). However, without better understanding of the underlying mechanisms or specific virologic interactions we cannot speculate whether the same association could apply to other emerging heterologous or hetero-subtypic variants; the antigenic distance and other criteria required to define or forecast that likelihood remain unknown.

In summary, although these ferret findings cannot be considered conclusive in explaining earlier human observations from Canada, they support the hypothesis that prior receipt of 2008–09 TIV may have had direct, adverse effects on A(H1N1)pdm09 illness. Both human and ferret findings from Canada are consistent with observations elsewhere of enhanced disease following heterologous influenza challenge in vaccinated swine. Given the potential implications for informing influenza immuno-epidemiology and public health response to other emerging viruses, these signals warrant further in-depth evaluation and a search for possible mechanistic explanations.

## Supporting Information

Figure S1 **HA1 microarray values for study and non-study antigens by group and study day.**
(PDF)

Table S1 **Amino acid sequence substitutions in hemagglutinin (HA) and neuraminidase (NA) of MDCK-passaged assay and challenge viruses.**
(PDF)

Table S2 **Pairwise identity (% (number of amino acid mutations)) in influenza A hemagglutinin 1 (HA1) peptide and antigenic sites across viruses used in antibody assays and A(H1N1)pdm09 challenge.**
(PDF)

Table S3 **Recombinant proteins of the HA1 part of the hemagglutinin (HA) protein of study and non-study viruses used in the protein microarray assay.**
(PDF)

Table S4 **Individual ferret haemagglutination inhibition (HI), microneutralization (MN) and ELISA (E) antibody titers among animals sacrificed at day 54 (Ch+5) with percent weight loss at Ch+5.**
(PDF)

Table S5 **Individual ferret haemagglutination inhibition (HI), microneutralization (MN) and ELISA (E) antibody titers among animals sacrificed at day 63 (Ch+14) with percent weight loss at Ch+5, Vaccinated Group.**
(PDF)

Table S6 **Individual ferret haemagglutination inhibition (HI), microneutralization (MN) and ELISA (E) antibody titers among animals sacrificed at day 63 (Ch+14) with percent weight loss at Ch+5, Placebo Group.**
(PDF)

Table S7 **Summary haemagglutination inhibition results by time, study group and antigen.**
(PDF)

Table S8 **Summary microneutralization results by time, study group and antigen.**
(PDF)

Table S9 **Individual ferret lung histopathology scores at sacrifice days 5 (Ch+5) or 14 (Ch+14) post-challenge and % weight loss from baseline at Ch+5.**
(PDF)
