# Supplementary material for: Randomized Controlled Ferret Study to Assess the Direct Impact of 2008–09 Trivalent Inactivated Influenza Vaccine on A(H1N1)pdm09 Disease Risk
Source: PLoS One. 2014 Jan 27;9(1):e86555. doi: 10.1371/journal.pone.0086555 (PMC3903544; doi:10.1371/journal.pone.0086555)
Supplement: Figure S1 — HA1 microarray values for study and non-study antigens by group and study day. (PDF) [file pone.0086555.s001.pdf]

Figure S1. HA1 microarray values for study and non-study antigens by group and study day

A) Pre-immunization

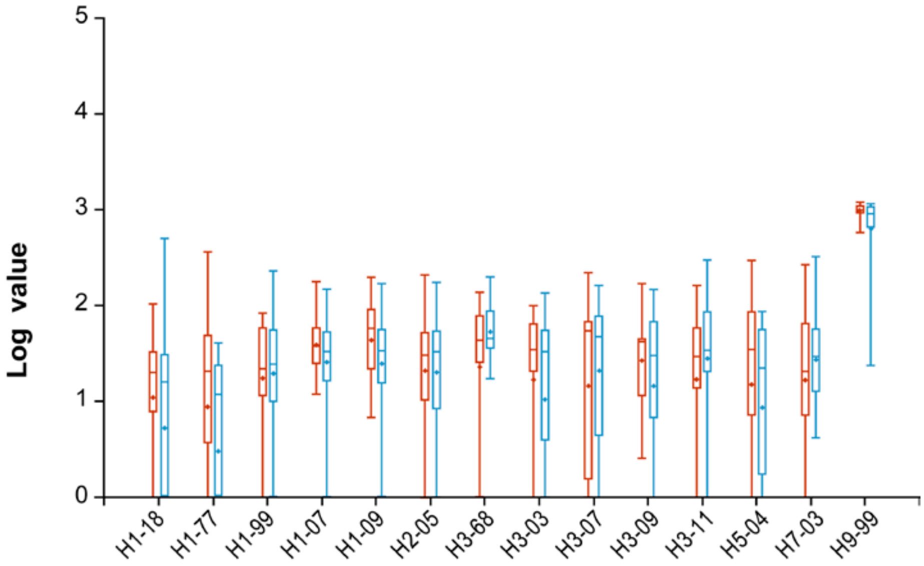

B) Day 28

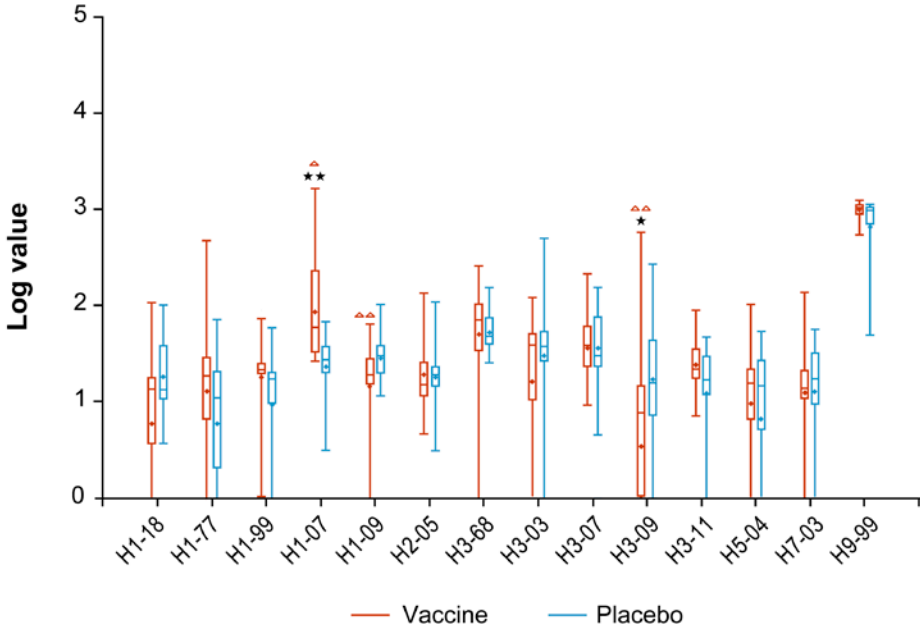

Box plots display median (dash) and mean (dot) of log-transformed HA1 immuno-blot antibody signal values for study and non-study antigens at (A) pre-immunization; (B) day 28; and overleaf (C) day 49 (Ch0); and (D) day 63 (Ch+14). The box extends to the 25th/75th percentiles and whiskers extend to minimum/maximum values. Antigens are as indicated in **Table S3**. Sample size as follows: Pre-immunization Vaccine=15, Placebo=16 (3 ferrets each per group pre-shipment serum substituted owing to insufficient day 0 available); Day 28 Vaccine=14, Placebo=15. ** indicates statistical significance at $p<0.01$ and * indicates statistical significance at $p<0.05$ in comparing vaccine to placebo group at the designated time point. ΔΔ indicates statistical significance at $p<0.01$ and Δ indicates statistical significance at $p<0.05$ in comparing day 28 to pre-immunization (panel B) within groups, colour coded by vaccine (red) or placebo (blue).

Figure S1. HA1 microarray values for study and non-study antigens by group and study day

C) Day 49 (Ch0)

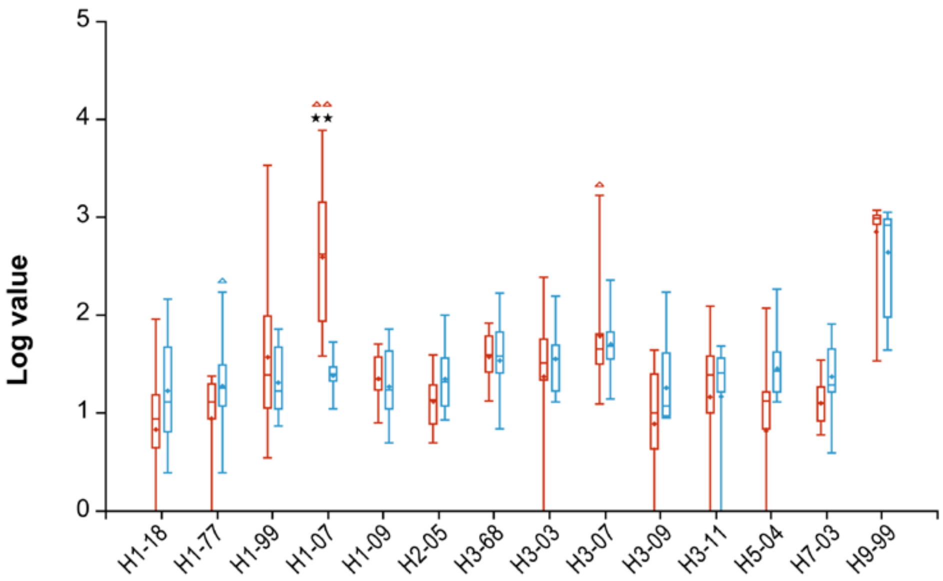

D) Day 63 (Ch+14)

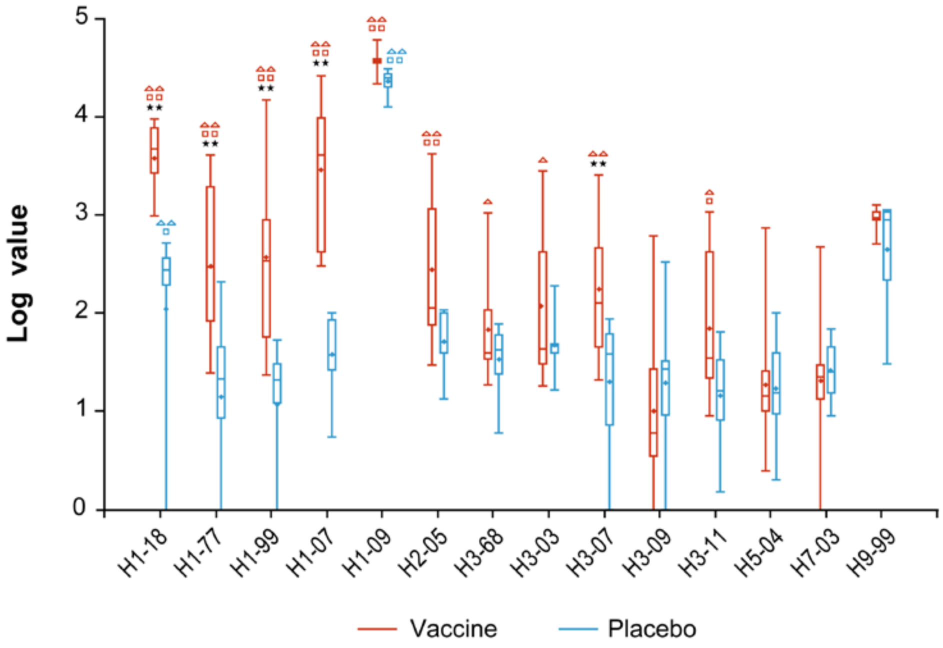

Box plots display median (dash) and mean (dot) of log-transformed HA1 immuno-blot antibody signal values for study and non-study antigens at (C) day 49 (Ch0) and (D) day 63 (Ch+14). The box extends to the $25^{th}/75^{th}$ percentiles and whiskers extend to minimum/maximum values. Antigens are as indicated in **Table S3**. Sample size as follows: Day 49 Vaccine=12, Placebo=11; Day 63 Vaccine=9, Placebo=8. ** indicates statistical significance at $p<0.01$ and * indicates statistical significance at $p<0.05$ in comparing vaccine to placebo group at the designated time point. ΔΔ indicates statistical significance at $p<0.01$ and Δ indicates statistical significance at $p<0.05$ in comparing day 49 (panel C) or day 63 (panel D) to pre-immunization within groups, colour coded by vaccine (red) or placebo (blue). □□ indicates statistical significance at $p<0.01$ and □ indicates statistical significance at $p<0.05$ in comparing day 63 to day 49 within groups, colour coded per above by study group.
