# Supplementary material for: Randomized Controlled Ferret Study to Assess the Direct Impact of 2008–09 Trivalent Inactivated Influenza Vaccine on A(H1N1)pdm09 Disease Risk
Source: PLoS One. 2014 Jan 27;9(1):e86555. doi: 10.1371/journal.pone.0086555 (PMC3903544; doi:10.1371/journal.pone.0086555)
Supplement: Table S1 — Amino acid sequence substitutions in hemagglutinin (HA) and neuraminidase (NA) of MDCK-passaged assay and challenge viruses. (PDF) [file pone.0086555.s002.pdf]

Table S1. Amino acid sequence substitutions in hemagglutinin (HA) and neuraminidase (NA) of MDCK-passaged assay and challenge viruses with affected antigenic sites indicated in parentheses relative to comparator strains

A. Comparator strains: posted references strains recommended by the WHO[a] for use in the 2008-09 trivalent influenza vaccine

Viruses used in antibody assays	Passage[b] Number	Haemagglutination Inhibition Assay		Microneutralization Assay	
		HA	NA	HA	NA
A/Brisbane/59/2007(H1N1)-like (Seasonal H1N1)	2	Asp/Ile186Asn (Sb)[c]	None[d]	Asp/Ile186Asn (Sb) Asp438Asn[e]	None[d]
A/Brisbane/10/2007(H3N2)-like[f] (Seasonal H3N2)	3	Asn96Ser (D) Leu194Pro (B)	None	Asn96Ser (D) Leu194Pro (B)	None
B/Florida/4/2006-like (Influenza B)	2	None[g]	None[h]	Mix Gly414Asp[i]	None[h]
A/California/7/2009-like[j] A(H1N1)pdm09 (WHO reference)	3	Ser183Pro	None	Ser183Pro	None
A/Quebec/144147/2009(H1N1)[k] A(H1N1)pdm09 (challenge virus)	3	Pro83Ser Ser203Thr (Ca1) Ile321Val	Val106Ile Asn248Asp	Pro83Ser Ser203Thr (Ca1) Ile321Val	Val106Ile Asn248Asp

B. Comparator strains: egg-passaged reassortant influenza A/H1N1[l] and A/H3N2[m] vaccine strains actually used by manufacturers in the 2008-09 trivalent influenza vaccine

Viruses used in antibody assays	Passage[b] Number	Haemagglutination Inhibition Assay		Microneutralization Assay	
		HA	NA	HA	NA
A/Brisbane/59/2007(H1N1)-like (Seasonal H1N1)	2	None[c]	None[d]	Asp438Asn[e]	None[d]
A/Brisbane/10/2007(H3N2)-like[f] (Seasonal H3N2)	3	Asn96Ser (D) Ser138Ala (A)[n] Gly142Arg (A)[n]	Thr26Ile[n] Ile32Thr[n] His150Arg[n] Ile267Thr[n] Ile312Thr[n]	Asn96Ser (D) Ser138Ala (A)[n] Gly142Arg (A)[n]	Thr26Ile[n] Ile32Thr[n] His150Arg[n] Ile267Thr[n] Ile312Thr[n]

Note: Amino acid numbering begins at first neuraminidase methionine for NA and after removal of the signal peptide for HA (residues 1-16 for H3, 1-17 for H1 and 1-15 for influenza B).

a. GenBank/GISAID Accession Numbers: CY035022, CY035024, CY030230, EPI162332, CY033876, CY033878, FJ981613, FJ984386
b. Passaged in Madin Darby canine kidney (MDCK) cells
c. GenBank Accession Number KF009550
d. GenBank Accession Number CY065749
e. GenBank Accession Number CY065747
f. GenBank Accession Numbers CY065751 (HA) and CY065752 (NA)
g. GenBank Accession Number KF009552
h. GenBank Accession Number CY073896
i. GenBank Accession Number CY073895
j. GenBank Accession Numbers KF009554-KF009555
k. GenBank Accession Numbers FN434457-FN434464
l. A/Brisbane/59/2007 (H1N1) – IVR-148
m. A/Uruguay/716/2007 (H3N2) – X-175C
n. Differences due to manufacturer vaccine virus strain selection rather than passage of WHO reference virus used in antibody assays.

Summary: For seasonal H1N1 there was 100% HA and NA identity between assay and vaccine (IVR-148) viruses. For A(H1N1)pdm09 there was a single AA HA antigenic site and 2AA NA difference between assay and challenge viruses. For H3N2 the MDCK-passaged virus showed 2AA HA antigenic site and no NA differences from the WHO-recommended reference virus but 3AA HA antigenic site differences from the X-175C vaccine component actually used by manufacturers. However, the X-175C vaccine component itself differed from the WHO-recommended reference by 3AA in the HA and 5AA in the NA. See **Table S2** for percent pairwise identity calculations for HA1 and antigenic sites.
