# Supplementary material for: Randomized Controlled Ferret Study to Assess the Direct Impact of 2008–09 Trivalent Inactivated Influenza Vaccine on A(H1N1)pdm09 Disease Risk
Source: PLoS One. 2014 Jan 27;9(1):e86555. doi: 10.1371/journal.pone.0086555 (PMC3903544; doi:10.1371/journal.pone.0086555)
Supplement: Table S2 — Pairwise identity (% (number of amino acid mutations)) in influenza A hemagglutinin 1 (HA1) peptide and antigenic sites across viruses used in antibody assays and A(H1N1)pdm09 challenge. (PDF) [file pone.0086555.s003.pdf]

Table S2. Pairwise identity (%[a] (number of amino acid mutations)) in influenza A hemagglutinin 1 (HA1) peptide and antigenic sites across viruses used in antibody assays and A(H1N1)pdm09 challenge

**H3 Subtype** (*Antigenic sites A-E comprising 131 amino acid residues[1])	**HA1**			**Antigenic Sites***		
	**A/Brisbane/10/2007 (reference)**	**A/Uruguay/716/2007 X-175C**	**Assay Brisbane/10/2007**	**A/Brisbane/10/2007 (reference)**	**A/Uruguay/716/2007 X-175C**	**Assay Brisbane/10/2007**
**A/Brisbane/10/2007 (reference)**	–	–	–	–	–	–
**A/Uruguay/716/2007 X-175C**	99.0 (3)	–	–	97.7 (3)	–	–
**Assay Brisbane/10/2007**	99.4 (2)	99.0 (3)	–	98.5 (2)	97.7 (3)	–

**H1 Subtype** (**Antigenic sites Cb, Sa, Ca2, Ca1, Sb comprising 50 amino acid residues[2])	**HA1**						**Antigenic Sites****					
	**A/Brisbane/59/2007 (reference)**	**Brisbane/59/2007 IVR-148**	**Assay Brisbane/59/2007**	**A/California/07/2009 (reference)**	**Assay A/California/07/2009**	**Challenge A/California/07/2009**	**A/Brisbane/59/2007 (reference)**	**Brisbane/59/2007 IVR-148**	**Assay Brisbane/59/2007**	**A/California/07/2009 (reference)**	**Assay A/California/07/2009**	**Challenge A/California/07/2009**
**A/Brisbane/59/2007 (reference)**	–	–	–	–	–	–	–	–	–	–	–	–
**A/Brisbane/59/2007 IVR-148**	99.7 (1)	–	–	–	–	–	98 (1)	–	–	–	–	–
**Assay Brisbane/59/2007**	99.7 (1)	100 (0)	–	–	–	–	98 (1)	100 (0)	–	–	–	–
**A/California/07/2009 (reference)**	72.8 (89)	72.5 (90)	72.5 (90)	–	–	–	50 (25)	48 (26)	48 (26)	–	–	–
**Assay A/California/07/2009**	73.1 (88)	72.8 (89)	72.8 (89)	99.7 (1)	–	–	50 (25)	48 (26)	48 (26)	100 (0)	–	–
**Challenge A/California/07/2009[¥]**	71.9 (92)	71.6 (93)	71.6 (93)	99.1 (3)	98.8 (4)	–	48 (26)	46 (27)	46 (27)	98 (1)	98 (1)	–

a. Percentage pairwise identity calculated as [1-(number of AA substitutions in HA1 or antigenic sites)/(total HA1 or antigenic site AA residues)]X100%

[1] Bush RM, Bender CA, Subbarao K, Cox NJ, Fitch WM. Predicting the evolution of human influenza A. Science 1999;286:1921-5.
[2] Brownlee, G.G & Fodor E. 2001 The predicted antigenicity of the hemagglutinin of the 1918 Spanish influenza pandemic suggests an avian origin. Phil. Trans. R. Soc. Lond. 356, 1871-1876.

[¥]A/Quebec/144147/2009(H1N1) [GenBank Accession Numbers FN434457-FN434464
