# Supplementary material for: Randomized Controlled Ferret Study to Assess the Direct Impact of 2008–09 Trivalent Inactivated Influenza Vaccine on A(H1N1)pdm09 Disease Risk
Source: PLoS One. 2014 Jan 27;9(1):e86555. doi: 10.1371/journal.pone.0086555 (PMC3903544; doi:10.1371/journal.pone.0086555)
Supplement: Table S3 — Recombinant proteins of the HA1 part of the hemagglutinin (HA) protein of study and non-study viruses used in the protein microarray assay. (PDF) [file pone.0086555.s004.pdf]

Table S3. Recombinant proteins of the HA1 part of the hemagglutinin (HA) protein of study (grey shaded) and non-study viruses used in the protein microarray assay

Name	Influenza virus strain	Manufacturer
H1-18	A/South Carolina/1/1918 (H1N1)	Immune Technology
H1-77	A/USSR/92/1977 (H1N1)	Immune Technology
H1-99	A/New Caledonia/20/1999 (H1N1)	Immune Technology
H1-07	A/Brisbane/59/2007 (H1N1)	Immune Technology
H1-09	A/California/06/2009 [A(H1N1)pdm09]	Immune Technology
H2-05	121808-#19-(052 and 053) (H2N2)	Immune Technology
H3-68	A/Aichi/2/1968 (H3N2)	Sino Biological
H3-03	A/Wyoming/3/2003 (H3N2)	Immune Technology
H3-07	A/Brisbane/10/2007 (H3N2)	Immune Technology
H3-09	A/Victoria/210/2009 (H3N2)	Immune Technology
H3-11	A/Victoria/361/2011 (H3N2)	Immune Technology
H5-04	A/Vietnam/1194/2004 (H5N1)	Immune Technology
H7-03	A/Chicken/Netherlands/1/2003 (H7N7)	Immune Technology
H9-99	A/Guinea fowl/Hong Kong/WF10/1999 (H9N2)	Immune Technology
