# Supplementary material for: Randomized Controlled Ferret Study to Assess the Direct Impact of 2008–09 Trivalent Inactivated Influenza Vaccine on A(H1N1)pdm09 Disease Risk
Source: PLoS One. 2014 Jan 27;9(1):e86555. doi: 10.1371/journal.pone.0086555 (PMC3903544; doi:10.1371/journal.pone.0086555)
Supplement: Table S4 — Individual ferret haemagglutination inhibition (HI), microneutralization (MN) and ELISA (E) antibody titers among animals sacrificed at day 54 (Ch+5) with percent weight loss at Ch+5. (PDF) [file pone.0086555.s005.pdf]

Table S4. Individual ferret haemagglutination inhibition (HI), microneutralization (MN) and ELISA (E) antibody titers* among animals sacrificed at day 54 (Ch+5) with percent weight loss at Ch+5

	VACCINATED FERRETS												PLACEBO FERRETS											
Ferret ID:	63 (Lung Score: 0.5) [% wt loss = 3.7]			69 (Lung Score: 11.5) [% wt loss = 7.8]			80 (Lung Score: 1) [%wt loss = 6.0]			82 (Lung Score: 10) [%wt loss = 10.0]			58 (Lung Score:4) [% wt loss = 7.7]			60 (Lung Score: 3) [% wt loss = 4.8]			81 (Lung Score: 0.5) [% wt loss = 4.9]			84 (Lung Score: 1) [% wt loss = 1.1]		
Antibody Assay:	HI	MN	E	HI	MN	E	HI	MN	E	HI	MN	E	HI	MN	E	HI	MN	E	HI	MN	E	HI	MN	E
**Pre-Shipment**																								
sH1N1	5			5			5			5			5			5			5			5		
sH3N2	5	NA	1.2	5	NA	1.1	5	NA	1.5	5	NA	1.4	5	NA	1.1	5	NA	1.2	5	NA	1.5	5	NA	0.84
Influenza B	5			5			5			5			5			5			5			5		
A(H1N1)pdm09	5			5			5			5			5			5			5			5		
**Day 0**																								
sH1N1	14.1	5		10	NA		5	5		5	5		5	5		5	5		5	5		NA	NA	
sH3N2	5	5	1.0	5	5	1.0	5	5	1.0	5	5	1.1	5	5	1.0	5	5	1.1	5	5	1.0	NA	NA	NA
Influenza B	5	5		5	5		5	5		5	5		5	5		5	5		5	5		NA	NA	
A(H1N1)pdm09	5	5		5	NA		5	5		5	5		5	5		5	5		5	5		NA	NA	
**Day 28**																								
sH1N1	5	5		40	57		5	5		5	5		5	5		7	5		5	5		7	5	
sH3N2	5	5		80	113		5	5		5	5		10	5		5	5		5	5		5	5	
Influenza B	NA	NA	NA	NA	NA	NA	NA	NA	NA	NA	NA	NA	NA	NA	NA	NA	NA	NA	NA	NA	NA	NA	NA	NA
A(H1N1)pdm09	5	5		5	5		5	5		5	5		5	5		5	5		5	5		5	5	
**Day 49/Ch0**																								
sH1N1	10	5		28	57		10	5		5	5		5	5		5	5		5	5		160**	5	
sH3N2	5	5	0.4	80	113	0.2	5	5	0.3	5	5	0.3	5	5	0.9	5	5	1.0	5	5	1.0	5	5	1.0
Influenza B	5	5		5	5		5	5		5	5		5	5		5	5		5	5		160**	NA	
A(H1N1)pdm09	5	5		5	5		5	5		5	5		5	5		5	5		5	5		5	5	
**Day 54/Ch+5**																								
sH1N1	80	5		40	57		5	5		5	5		10	5		20	5		10	5		10	5	
sH3N2	10	5	0.3	80	80	0.1	5	5	0.2	5	5	0.2	5	5	1.1	5	5	1.1	5	5	0.9	5	5	1.0
Influenza B	NA	NA		NA	NA		NA	NA		NA	NA		NA	NA		NA	NA		NA	NA		NA	NA	
A(H1N1)pdm09	5	20		5	7.1		5	10		5	5		5	10		5	5		5	10		5	7.1	

Lung score = Combined lung inflammatory score at sacrifice Ch+5; % wt loss = percentage weight loss from baseline at Ch+5 (the study day with the greatest between-group difference in % weight loss); Ch=challenge; NA=Not available
sH1N1= seasonal H1N1=A/Brisbane/59/2007(H1N1)-like
sH3N2= seasonal H3N2=A/Brisbane/10/2007(H3N2)-like
Influenza B=B/Florida/4/2006(like)-like
A(H1N1)pdm09=A/California/7/2009-like
Titerls <10 assigned a value of 5.
ELISA values <0.60 considered positive; values ≥ 0.60 negative
*Geometric mean titer of duplicate HI and MN values displayed
**In the context of surrounding values and given that influenza B is not an animal pathogen, these titers are interpreted as non-specific reactivity. On repeat, both titers were 40 or less but there remained insufficient sera for further dilutional clarification. In deriving summary statistics, these values were therefore retained.
