# Supplementary material for: Randomized Controlled Ferret Study to Assess the Direct Impact of 2008–09 Trivalent Inactivated Influenza Vaccine on A(H1N1)pdm09 Disease Risk
Source: PLoS One. 2014 Jan 27;9(1):e86555. doi: 10.1371/journal.pone.0086555 (PMC3903544; doi:10.1371/journal.pone.0086555)
Supplement: Table S5 — Individual ferret haemagglutination inhibition (HI), microneutralization (MN) and ELISA (E) antibody titers among animals sacrificed at day 63 (Ch+14) with percent weight loss at Ch+5, Vaccinated Group. (PDF) [file pone.0086555.s006.pdf]

Table S5. Individual ferret haemagglutination inhibition (HI), microneutralization (MN) and ELISA (E) antibody titers* among animals sacrificed at day 63 (Ch+14) with percent weight loss at Ch+5, Vaccinated Group

Ferret ID:	51 [% wt loss = 10.6]			54 [% wt loss = 7.3]			56 [% wt loss = 9.8]			57 [% wt loss = 5.4]		
Assay:	HI	MN	E	HI	MN	E	HI	MN	E	HI	MN	E
**Pre-Shipment**												
sH1N1	5	NA	1.5	5	NA	1.1	5	NA	1.2	5	NA	1.0
H3N2	5	NA		5	NA		5	NA		5	NA	
Influenza B	5	NA		5	NA		5	NA		5	NA	
A(H1N1)pdm09	5	NA		5	NA		5	NA		5	NA	
**Day 0**												
sH1N1	10	5	1.3	5	5	1.0	5	5	1.0	5	5	1.0
H3N2	5	5		5	5		5	5		5	5	
Influenza B	5	5		5	5		5	5		5	5	
A(H1N1)pdm09	5	5		5	5		5	5		5	5	
**Day 28**												
sH1N1	7	5	NA	5	5	NA	5	5	NA	5	5	NA
H3N2	5	5		5	5		5	5		5	5	
Influenza B	NA	NA		NA	NA		NA	NA		NA	NA	
A(H1N1)pdm09	5	5		5	5		5	5		5	5	
**Day 49/Ch0**												
sH1N1	10	14	0.9	5	5	0.3	28	80	0.4	10	28	0.5
H3N2	10	7		5	5		20	7		10	5	
Influenza B	5	5		5	5		5	10		5	5	
A(H1N1)pdm09	5	5		5	5		5	5		5	5	
**Day 63/Ch+14**												
sH1N1	10	20	0.5	NA	NA	0.4	20	80	0.1	5	14	0.2
H3N2	20	40		NA	NA		10	20		5	7	
Influenza B	NA	NA		NA	NA		NA	NA		NA	NA	
A(H1N1)pdm09	2560	7241		NA	NA		2560	7240		2560	7240	

Ferret ID:	59 [% wt loss = 7.9]			61 [% wt loss = 8.3]			62 [% wt loss = 4.6]			65 [% wt loss = 9.8]		
Assay:	HI	MN	E	HI	MN	E	HI	MN	E	HI	MN	E
**Pre-Shipment**												
sH1N1	5	NA	1.2	5	NA	1.2	5	NA	1.1	5	NA	1.1
H3N2	5	NA		5	NA		5	NA		5	NA	
Influenza B	5	NA		5	NA		5	NA		5	NA	
A(H1N1)pdm09	5	NA		5	NA		5	NA		5	NA	
**Day 0**												
sH1N1	5	5	1.1	5	5	1.0	10	NA	NA	5	5	1.0
H3N2	5	5		5	5		5	5		5	5	
Influenza B	5	5		5	5		5	NA		5	5	
A(H1N1)pdm09	5	5		5	5		5	5		5	5	
**Day 28**												
sH1N1	10	10	NA	10	5	NA	40	80	NA	7	10	NA
H3N2	5	5		5	5		80	80		10	7	
Influenza B	NA	NA		NA	NA		NA	NA		NA	NA	
A(H1N1)pdm09	5	5		5	5		5	5		5	5	
**Day 49/Ch0**												
sH1N1	10	14	0.2	5	5	0.9	80	226	0.4	10	10	0.4
H3N2	5	10		5	5		160	453		10	20	
Influenza B	5	5		5	10		5	160		5	5	
A(H1N1)pdm09	5	5		5	5		5	5		5	5	
**Day 63/Ch+14**												
sH1N1	7	14	0.1	5	5	0.2	80	160	0.1	10	20	0.1
H3N2	5	7		5	5		5	226		10	14	
Influenza B	NA	NA		NA	NA		NA	NA		NA	NA	
A(H1N1)pdm09	2560	7240		2560	7240		1280	3620		2560	7240	

Ferret ID:	68 [% wt loss = 6.5]			70 [% wt loss = 2.4]			72 [% wt loss = 10.5]			85 [% wt loss = 12.1]		
Assay:	HI	MN	E	HI	MN	E	HI	MN	E	HI	MN	E
**Pre-Shipment**												
sH1N1	5	NA	1.0	5	NA	1.1	5	NA	1.1	5	NA	1.1
H3N2	5	NA		5	NA		5	NA		5	NA	
Influenza B	5	NA		5	NA		5	NA		5	NA	
A(H1N1)pdm09	5	NA		5	NA		5	NA		5	NA	
**Day 0**												
sH1N1	10	5	1.0	5	5	1.0	5	5	1.1	5	5	1.0
H3N2	5	5		5	5		5	5		5	5	
Influenza B	5	5		5	5		5	5		5	5	
A(H1N1)pdm09	5	5		5	5		5	5		5	5	
**Day 28**												
sH1N1	5	5	NA	5	5	NA	5	5	NA	5	5	NA
H3N2	5	7		5	5		5	5		5	5	
Influenza B	NA	NA		NA	NA		NA	NA		NA	NA	
A(H1N1)pdm09	5	5		5	5		5	5		5	5	
**Day 49/Ch0**												
sH1N1	5	5	1.0	5	5	0.69	NA	5	NA	NA	NA	NA
H3N2	5	14		5	5		NA	5		NA	NA	
Influenza B	5	5		5	5		NA	NA		NA	NA	
A(H1N1)pdm09	5	5		5	5		NA	NA		NA	NA	
**Day 63/Ch+14**												
sH1N1	5	5	0.1	NA	5	NA	10	7	0.2	5	5	0.2
H3N2	5	5		NA	5		10	20		5	14	
Influenza B	NA	NA		NA	NA		NA	NA		NA	NA	
A(H1N1)pdm09	5120	5120		NA	20480		1280	1280		1810	1280	

% wt loss = percentage weight loss from baseline at Ch+5 (the study day with the greatest between-group difference in % weight loss); Ch=challenge; NA=Not available
sH1N1= seasonal H1N1=A/Brisbane/59/2007(H1N1)-like
sH3N2= seasonal H3N2=A/Brisbane/10/2007(H3N2)-like
Influenza B=B/Florida/4/2006(like)-like
A(H1N1)pdm09=A/California/7/2009-like
Titerls <10 assigned a value of 5.
ELISA values <0.60 considered positive; values ≥ 0.60 negative
*Geometric mean titer of duplicate HI and MN values displayed
