# Supplementary material for: Randomized Controlled Ferret Study to Assess the Direct Impact of 2008–09 Trivalent Inactivated Influenza Vaccine on A(H1N1)pdm09 Disease Risk
Source: PLoS One. 2014 Jan 27;9(1):e86555. doi: 10.1371/journal.pone.0086555 (PMC3903544; doi:10.1371/journal.pone.0086555)
Supplement: Table S6 — Individual ferret haemagglutination inhibition (HI), microneutralization (MN) and ELISA (E) antibody titers among animals sacrificed at day 63 (Ch+14) with percent weight loss at Ch+5, Placebo Group. (PDF) [file pone.0086555.s007.pdf]

Table S6. Individual ferret haemagglutination inhibition (HI), microneutralization (MN) and ELISA (E) antibody titers among animals sacrificed at day 63 (Ch+14) with percent weight loss at Ch+5, Placebo Group

Ferret ID:	52 [% wt loss = 4.1]			53 [% wt loss = 5.4]			55 [% wt loss = 5.2]			64 [% wt loss = 7.1]		
Assay:	HI	MN	E	HI	MN	E	HI	MN	E	HI	MN	E
**Pre-Shipment**												
sH1N1	5	NA	1.1	5	NA	1.1	5	NA	1.2	5	NA	1.2
H3N2	5	NA		5	NA		5	NA		5	NA	
Influenza B	5	NA		5	NA		5	NA		5	NA	
A(H1N1)pdm09	5	NA		5	NA		5	NA		5	NA	
**Day 0**												
sH1N1	5	5	1.1	5	5	1.0	20	5	1.2	5	5	1.0
H3N2	5	5		5	5		5	5		5	5	
Influenza B	5	5		5	5		5	5		5	5	
A(H1N1)pdm09	5	5		5	5		5	5		5	5	
**Day 28**												
sH1N1	5	5	NA	10	5	NA	5	5	NA	5	5	NA
H3N2	5	5		5	5		5	5		5	5	
Influenza B	NA	NA		NA	NA		NA	NA		NA	NA	
A(H1N1)pdm09	5	5		5	5		5	5		5	5	
**Day 49/Ch0**												
sH1N1	5	5	1.0	5	5	1.0	5	5	1.0	5	5	1.0
H3N2	5	5		5	5		5	5		5	5	
Influenza B	5	5		5	5		5	5		5	5	
A(H1N1)pdm09	5	5		5	5		5	5		5	5	
**Day 63/Ch+14**												
sH1N1	5	5	0.5	5	5	0.61	10	5	0.5	5	5	0.5
H3N2	5	5		5	5		5	5		5	5	
Influenza B	NA	NA		NA	NA		NA	NA		NA	NA	
A(H1N1)pdm09	5120	7240		2560	7240		5120	20480		2560	14480	

Ferret ID:	66 [% wt loss = 4.4]			67 [% wt loss = 1.8]			71 [% wt loss = 1.1]			73 [% wt loss = 7.2]		
Assay:	HI	MN	E	HI	MN	E	HI	MN	E	HI	MN	E
**Pre-Shipment**												
sH1N1	5	NA	1.0	5	NA	1.1	5	NA	1.0	5	NA	1.1
H3N2	5	NA		5	NA		5	NA		5	NA	
Influenza B	5	NA		5	NA		5	NA		5	NA	
A(H1N1)pdm09	5	NA		5	NA		5	NA		5	NA	
**Day 0**												
sH1N1	7	5	1.0	10	5	1.1	5	5	1.0	5	5	1.1
H3N2	5	5		5	5		5	5		5	5	
Influenza B	5	5		5	5		5	5		5	5	
A(H1N1)pdm09	5	5		5	5		5	5		5	5	
**Day 28**												
sH1N1	5	5	NA	5	5	NA	5	5	NA	5	5	NA
H3N2	5	5		5	5		5	5		5	5	
Influenza B	NA	NA		NA	NA		NA	NA		NA	NA	
A(H1N1)pdm09	5	5		5	5		5	5		5	5	
**Day 49/Ch0**												
sH1N1	5	5	1.0	5	5	1.0	NA	5	NA	5	5	1.1
H3N2	5	5		5	5		NA	5		5	5	
Influenza B	5	5		5	5		NA	NA		5	5	
A(H1N1)pdm09	5	5		5	5		NA	5		5	5	
**Day 63/Ch+14**												
sH1N1	5	5	0.4	NA	NA	NA	5	5	0.4	5	5	0.3
H3N2	5	5		NA	NA		5	5		5	5	
Influenza B	NA	NA		NA	NA		NA	NA		NA	NA	
A(H1N1)pdm09	1810	14480		NA	NA		2560	3620		2560	3620	

Ferret ID:	75 [% wt loss = 5.3]			77 [% wt loss = 11.3]			78 [% wt loss = 3.9]			79 [% wt loss = 7.2]		
Assay:	HI	MN	E	HI	MN	E	HI	MN	E	HI	MN	E
**Pre-Shipment**												
sH1N1	5	NA	1.1	5	NA	1.4	5	NA	1.5	5	NA	1.4
H3N2	5	NA		5	NA		5	NA		5	NA	
Influenza B	5	NA		5	NA		5	NA		5	NA	
A(H1N1)pdm09	5	NA		5	NA		5	NA		5	NA	
**Day 0**												
sH1N1	5	5	1.1	5	5	1.0	5	5	1.0	5	5	1.0
H3N2	5	5		5	5		5	5		5	5	
Influenza B	5	5		5	5		5	5		5	5	
A(H1N1)pdm09	5	5		5	5		5	5		5	5	
**Day 28**												
sH1N1	5	5	NA	5	5	NA	5	5	NA	5	5	NA
H3N2	5	5		5	5		5	5		5	5	
Influenza B	NA	NA		NA	NA		NA	NA		NA	NA	
A(H1N1)pdm09	5	5		5	5		5	5		5	5	
**Day 49/Ch0**												
sH1N1	28	5	NA	NA	NA	NA	5	5	1.1	7	5	1.1
H3N2	5	5		NA	NA		5	5		5	5	
Influenza B	5	5		NA	NA		5	5		5	5	
A(H1N1)pdm09	5	5		NA	NA		5	5		5	5	
**Day 63/Ch+14**												
sH1N1	5	5	0.61	7	5	0.4	5	5	0.4	5	5	0.4
H3N2	5	5		5	5		5	5		5	5	
Influenza B	NA	NA		NA	NA		NA	NA		NA	NA	
A(H1N1)pdm09	3620	3620		3620	5120		2560	2560		2560	5120	

% wt loss = percentage weight loss from baseline at Ch+5 (the study day with the greatest between-group difference in % weight loss); Ch=challenge; NA=Not available
sH1N1= seasonal H1N1=A/Brisbane/59/2007(H1N1)-like
sH3N2= seasonal H3N2=A/Brisbane/10/2007(H3N2)-like
Influenza B=B/Florida/4/2006(like)-like
A(H1N1)pdm09=A/California/7/2009-like
Titerls <10 assigned a value of 5.
ELISA values <0.60 considered positive; values ≥ 0.60 negative
*Geometric mean titer of duplicate HI and MN values displayed
