# Supplementary material for: Randomized Controlled Ferret Study to Assess the Direct Impact of 2008–09 Trivalent Inactivated Influenza Vaccine on A(H1N1)pdm09 Disease Risk
Source: PLoS One. 2014 Jan 27;9(1):e86555. doi: 10.1371/journal.pone.0086555 (PMC3903544; doi:10.1371/journal.pone.0086555)
Supplement: Table S7 — Summary haemagglutination inhibition results by time, study group and antigen. (PDF) [file pone.0086555.s008.pdf]

Table S7. Summary haemagglutination inhibition results by time, study group and antigen

Time Point (N per group), Influenza antigens [a,b,c,d]	Titer ≥40		Titer ≥10		GMT (95%CI)		Sero-conversion[e]		GMTR[e]	
	Vaccine n	Placebo n	Vaccine n	Placebo n	Vaccine n	Placebo n	Vaccine n	Placebo n	Vaccine n	Placebo n
**Pre-Shipment (16)**	**N=16**	**N=16**	**N=16**	**N=16**	**N=16**	**N=16**	**NA**	**NA**	**NA**	**NA**
Seasonal H1N1	0	0	0	0	5	5	NA	NA	NA	NA
Seasonal H3N2	0	0	0	0	5	5	NA	NA	NA	NA
Influenza B	0	0	0	1	5	5	NA	NA	NA	NA
A(H1N1)pdm09	0	0	0	0	5	5	NA	NA	NA	NA
**Day 0 (16)**	**N=16**	**N=15[f]**	**N=16**	**N=15[f]**	**N=16**	**N=15**	**NA**	**NA**	**NA**	**NA**
Seasonal H1N1	0	0	5	2	6.3 (5.2-7.7)	5.9 (4.7-.3)	NA	NA	NA	NA
Seasonal H3N2	0	0	0	0	5	5	NA	NA	NA	NA
Influenza B	0	0	0	0	5	5	NA	NA	NA	NA
A(H1N1)pdm09	0	0	0	0	5	5	NA	NA	NA	NA
**Day 28 (16)**	**N=16**	**N=16**	**N=16**	**N=16**	**N=16**	**N=16**	**N=16**	**N=16[f]**	**N=16**	**N=16[f]**
Seasonal H1N1	2	0	4	1	7.4 (5.1-10.7)	5.5 (4.9-6.1)	2	0	1.16	0.94
Seasonal H3N2	2	0	3	1	7.4 (4.5-12.2)	5.2 (4.8-5.7)	2	0	1.48	1.04
Influenza B	ND	ND	ND	ND	ND	ND	ND	ND	ND	ND
A(H1N1)pdm09	0	0	0	0	5	5	0	0	1.00	1.00
**Day 49/Ch0 (16)**	**N=14**	**N=14**	**N=14**	**N=14**	**N=14**	**N=14**	**N=14**	**N=14[f]**	**N=14**	**N=14[f]**
Seasonal H1N1	1	1	9	2	10.5 (6.5-16.9)	7.4 (4.2-13.2)	1	0	1.66	1.28
Seasonal H3N2	2	0	6	0	10.0 (5.2-19.1)	5	2	0	2.00	1.00
Influenza B	0	1	0	1	5	6.4 (3.8-10.9)	0	0	1.00	1.28
A(H1N1)pdm09	0	0	0	0	5	5	0	0	1.00	1.00
**Day 54/Ch+5 (4)**	**N=4**	**N=4**	**N=4**	**N=4**	**N=4**	**N=4**	**N=4**	**N=4[f]**	**N=4**	**N=4[f]**
Seasonal H1N1	2	0	2	4	16.8 (1.7-163.4)	11.9 (6.9-20.6)	2	0	2.65	2.04
Seasonal H3N2	1	0	2	0	11.9 (1.5-95.9)	5	1	0	2.38	1.00
Influenza B	ND	ND	ND	ND	ND	ND	ND	ND	ND	ND
A(H1N1)pdm09	0	0	0	0	5	5	0	0	1.00	1.00
**Day 63/Ch+14 (12)**	**N=10**	**N=11**	**N=10**	**N=11**	**N=10**	**N=11**	**N=10**	**N=11**	**N=10**	**N=11**
Seasonal H1N1	1	0	5	1	9.7 (5.2-18.0)	5.5 (4.7-6.4)	1	0	1.52	0.93
Seasonal H3N2	0	0	4	0	7.1 (5.0-10.0)	5	1	0	1.41	1.00
Influenza B	ND	ND	ND	ND	ND	ND	ND	ND	ND	ND
A(H1N1)pdm09	10	11	10	11	2307 (1731-3076)	2997 (2411-3725)	10	11	461	599

Sero-conversion = number with four-fold or greater rise in geometric mean titer (GMT) or from titer <10 to at least 40. Titers <10 assigned a value of 5; NA=Not Applicable; ND= Not done; Ch=challenge; GMTR: GMT ratio relative to Day 0. Where numbers (N) differ from the number randomized per group it is because insufficient sera remained for testing of all animals.
a. Seasonal H1N1=A/Brisbane/59/2007(H1N1)-like; b. Seasonal H3N2=A/Brisbane/10/2007(H3N2)-like; c. Influenza B=B/Florida/4/2006-like; d. A(H1N1)pdm09=A/California/07/2009-like; e. Relative to Day 0; f. Missing pre-immunization (Day 0) serum for one placebo ferret substituted with pre-shipment serum for deriving sero-conversion and GMTR.
