# Supplementary material for: Randomized Controlled Ferret Study to Assess the Direct Impact of 2008–09 Trivalent Inactivated Influenza Vaccine on A(H1N1)pdm09 Disease Risk
Source: PLoS One. 2014 Jan 27;9(1):e86555. doi: 10.1371/journal.pone.0086555 (PMC3903544; doi:10.1371/journal.pone.0086555)
Supplement: Table S8 — Summary microneutralization results by time, study group and antigen. (PDF) [file pone.0086555.s009.pdf]

Table S8. Summary microneutralization results by time, study group and antigen

Time Point	Titer ≥40		Titer ≥10		GMT (95%CI)		Sero-conversion[e]		GMTR[e]	
(N per group), Influenza antigens[a,b,c,d]	Vaccine n	Placebo n	Vaccine n	Placebo n	Vaccine n	Placebo n	Vaccine n	Placebo n	Vaccine n	Placebo n
**Day 0 (16)**	**N=14**	**N=15**	**N=14**	**N=15**	**N=14**	**N=15**	**NA**	**NA**	**NA**	**NA**
Seasonal H1N1	0	0	0	0	5	5	NA	NA	NA	NA
Seasonal H3N2	0[f]	0	0[f]	0	5[f]	5	NA	NA	NA	NA
Influenza B	0	0	0	0	5	5	NA	NA	NA	NA
A(H1N1)pdm09	0	0	0	0	5	5	NA	NA	NA	NA
**Day 28 (16)**	**N=16**	**N=16**	**N=16**	**N=16**	**N=16**	**N=16**	**N=14**	**N=15**	**N=14**	**N=15**
Seasonal H1N1	2	0	4	0	7.5 (4.7-12.1)	5	0	0	1.51	1.00
Seasonal H3N2	2	0	2	0	7.5 (4.4-12.8)	5	2[f]	0	1.51	1.00
Influenza B	ND	ND	ND	ND	ND	ND	ND	ND	ND	ND
A(H1N1)pdm09	0	0	0	0	5	5	0	0	1.00	1.00
**Day 49/Ch0 (16)**	**N=15**	**N=15**	**N=15**	**N=15**	**N=15**	**N=15**	**N=15**	**N=14**	**N=15**	**N=14**
Seasonal H1N1	3	0	7	0	12.3 (6.2-24.5)	5	1[g]	0	1.56[g]	1.00
Seasonal H3N2	2	0	5	0	10.7 (5.1-22.5)	5	2	0	2.14	1.00
Influenza B	1[g]	0	3[g]	0	7.1 (4.2-12.0)[g]	5	0[h]	0	1.22[h]	1.00
A(H1N1)pdm09	0[i]	0	0[i]	0	5[i]	5	0[h]	0	1.00[h]	1.00
**Day 54/Ch+5 (4)**	**N=4**	**N=4**	**N=4**	**N=4**	**N=4**	**N=4**	**N=4**	**N=3**	**N=4**	**N=3**
Seasonal H1N1	1	0	1	0	9.2 (1.3-63.2)	5	0[j]	0	1.83[j]	1.00
Seasonal H3N2	1	0	1	0	10.0 (1.1-90.8)	5	1	0	2.00	1.00
Influenza B	ND	ND	ND	ND	ND	ND	ND	ND	ND	ND
A(H1N1)pdm09	0	0	2	2	9.2 (3.6-23.5)	7.7 (4.5-13.1)	0[j]	0	1.83[j]	1.54
**Day 63/Ch+14 (12)**	**N=11**	**N=11**	**N=11**	**N=11**	**N=11**	**N=11**	**N=10**	**N=11**	**N=10**	**N=11**
Seasonal H1N1	2	0	6	0	14.1 (6.4-31.3)	5	1	0	2.83	1.00
Seasonal H3N2	2	0	6	0	14.1 (6.5-30.6)	5	2[k]	0	2.83[k]	1.00
Influenza B	ND	ND	ND	ND	ND	ND	ND	ND	ND	ND
A(H1N1)pdm09	11	11	11	11	5284 (3062-9118)	6383 (4041-10083)	10	11	1057	1277

Sero-conversion = number with four-fold or greater rise in geometric mean titer (GMT) or from titer <10 to at least 40. Titers <10 assigned a value of 5; NA=Not Applicable; ND= Not done; Ch=challenge; GMTR: GMT ratio relative to Day 0. Where numbers (N) differ from the number randomized per group it is because insufficient sera remained for testing of all animals.
a. Seasonal H1N1=A/Brisbane/59/2007(H1N1)-like; b. Seasonal H3N2=A/Brisbane/10/2007(H3N2)-like; c. Influenza B=B/Florida/4/2006-like; d. A(H1N1)pdm09=A/California/07/2009-like; e. Relative to Day 0; f. N=16; g. N=13; h. N=12; i. N=14; j. N=3; k. N=11;
