# Supplementary material for: Randomized Controlled Ferret Study to Assess the Direct Impact of 2008–09 Trivalent Inactivated Influenza Vaccine on A(H1N1)pdm09 Disease Risk
Source: PLoS One. 2014 Jan 27;9(1):e86555. doi: 10.1371/journal.pone.0086555 (PMC3903544; doi:10.1371/journal.pone.0086555)
Supplement: Table S9 — Individual ferret lung histopathology scores at sacrifice days 5 (Ch+5) or 14 (Ch+14) post-challenge and % weight loss from baseline at Ch+5. (PDF) [file pone.0086555.s010.pdf]

Table S9. Individual ferret lung histopathology scores at sacrifice days 5 (Ch+5) or 14 (Ch+14) post-challenge and % weight loss from baseline at Ch+5

Ferret ID	Group	Day Post-Challenge (Ch+5/14)	Inflammatory Indicators							Other Indicators		Lung virus titer (log pfu/mL)	% weight loss from baseline at Ch+5[a]
			Endo/ bronchial	Peri-bronchial	Peri-vascular	Inter stitial	Pleural	Intra alveolar	Combined Inflammatory	Vascluar Congestion	Pulmonary Edema		
63*	Vaccine	5	0	0	0.5	0	0	0	0.5	0.5	0	4.88	3.7
69*	Vaccine	5	2.5	2	1	2	1	3	11.5	1.5	1	5.25	7.8
80	Vaccine	5	0	1	0	0	0	0	1	0.5	0.5	4.70	6.0
82	Vaccine	5	3	3	1	1	0	2	10	2	1	5.02	10.0
58*	Placebo	5	2	1	0.5	0.5	0	0	4	1.5	1	3.95	7.7
60	Placebo	5	2	1	0	0	0	0	3	0.5	0	4.69	4.8
81*	Placebo	5	0	0.5	0	0	0	0	0.5	0.5	0	4.11	4.9
84	Placebo	5	0	0.5	0.5	0	0	0	1	0.5	0	4.17	1.1
51	Vaccine	14	0	0	0	0	0.5	0	0.5	0	0	0	10.6
54	Vaccine	14	0	1	0.5	1	0	0	2.5	0.5	0	0	7.3
56	Vaccine	14	0	1	0	1	0	0	2	1	0	0	9.8
57	Vaccine	14	0	0	0.5	0.5	0	0	1	1	0	0	5.4
59	Vaccine	14	0	1	1	0	0	0	2	0	0	0	7.9
61	Vaccine	14	0	1	1	1.5	0	0	3.5	0	0	0	8.3
62	Vaccine	14	0	0.5	0.5	0	0	0	1	0	0	0	4.6
65	Vaccine	14	0	1.5	1.5	1.5	0	0	4.5	0	0	0	5.4
68	Vaccine	14	0	0.5	0.5	0	0	0	1	0	0	0	6.5
70	Vaccine	14	0	0.5	0.5	0.5	0	0	1.5	0	0	0	2.4
72	Vaccine	14	0	0.5	0.5	0	0	0	1	0	0	0	10.5
85	Vaccine	14	0	0	0.5	0.5	0	0	1	0	0	0	12.1
52	Placebo	14	0	0	0.5	0	0	0	0.5	0.5	0	0	4.1
53	Placebo	14	0	0	0	0.5	0	0	0.5	0.5	0	0	5.4
55	Placebo	14	0	0	0	0	0	0	0	0	0	0	5.2
66	Placebo	14	1	1	1.5	0	0	0	3.5	0	0	0	4.4
67	Placebo	14	1.5	2	1	1	0	0	5.5	0	0	0	1.8
71	Placebo	14	0.5	0.5	0.5	0	0	0	1.5	0	0	0	1.1
73	Placebo	14	0	1	0.5	0.5	0	0	2	0	0	0	7.2
75	Placebo	14	0	0.5	0.5	0	0	0	1	0	0	0	5.3
78	Placebo	14	1	2	1.5	0.5	0	0	5	0	0	0	3.9
79	Placebo	14	0	0.5	0.5	0.5	0	0	1.5	0	0	0	7.2
64	Placebo	14	0	1	0	0.5	0	0	1.5	0	0	0	7.1
77	Placebo	14	0.5	2	2	2	0	0	6.5	0	0	0	11.3

Individual indicators scored from 0 (normal), 1 (mild), 2 (moderate) or 3 (marked) for a maximum combined inflammatory score of 18.
*Photo illustration of lung histo-pathology at Ch+5 (**Figure 4**)
a. The greatest between-group difference in percentage weight loss from baseline was at Ch+5, overall and among animals sacrificed at Ch+5 or Ch+14. Ch+5 weight loss is thus displayed for context (see text)
